# *CTNND1* variants cause familial exudative vitreoretinopathy through the Wnt/cadherin axis

**DOI:** 10.1172/jci.insight.158428

**Published:** 2022-07-22

**Authors:** Mu Yang, Shujin Li, Li Huang, Rulian Zhao, Erkuan Dai, Xiaoyan Jiang, Yunqi He, Jinglin Lu, Li Peng, Wenjing Liu, Zhaotian Zhang, Dan Jiang, Yi Zhang, Zhilin Jiang, Yeming Yang, Peiquan Zhao, Xianjun Zhu, Xiaoyan Ding, Zhenglin Yang

**Affiliations:** 1 Sichuan Provincial Key Laboratory for Human Disease Gene Study, Center for Medical Genetics, Sichuan Provincial People’s Hospital, University of Electronic Science and Technology of China, Chengdu, China.; 2 Research Unit for Blindness Prevention of the Chinese Academy of Medical Sciences (2019RU026), Sichuan Academy of Medical Sciences and Sichuan Provincial People’s Hospital, Chengdu, China.; 3 State Key Laboratory of Ophthalmology, Zhongshan Ophthalmic Center, Sun Yat-sen University, Guangzhou, China.; 4 Department of Ophthalmology, Xinhua Hospital Affiliated to Shanghai Jiaotong University School of Medicine, Shanghai, China.; 5 Key Laboratory of Tibetan Medicine Research and Qinghai Provincial Key Laboratory of Tibetan Medicine Research, Northwest Institute of Plateau Biology, Chinese Academy of Sciences, Xining, China.

**Keywords:** Genetics, Ophthalmology, Endothelial cells, Genetic diseases

## Abstract

Familial exudative vitreoretinopathy (FEVR) is a hereditary disorder that can cause vision loss. *CTNND1* encodes a cellular adhesion protein p120-catenin (p120), which is essential for vascularization with unclear function in postnatal physiological angiogenesis. Here, we applied whole-exome sequencing to 140 probands of FEVR families and identified 3 candidate variants in the human *CTNND1* gene. We performed inducible deletion of *Ctnnd1* in the postnatal mouse endothelial cells (ECs) and observed typical phenotypes of FEVR with reactive gliosis. Using unbiased proteomics analysis combined with experimental approaches, we conclude that p120 is critical for the integrity of adherens junctions (AJs) and that p120 activates Wnt signaling activity by protecting β-catenin from glycogen synthase kinase 3 beta–ubiqutin–guided (Gsk3β-ubiquitin–guided) degradation. Treatment of *CTNND1*-depleted human retinal microvascular ECs with Gsk3β inhibitors LiCl or CHIR-99021 enhanced cell proliferation. Moreover, LiCl treatment increased vessel density in *Ctnnd1*-deficient mouse retinas. Variants in *CTNND1* caused FEVR by compromising the expression of AJs and Wnt signaling activity. Genetic interactions between p120 and β-catenin or α-catenin revealed by double-heterozygous deletion in mice showed that p120 regulates vascular development through the Wnt/cadherin axis. In conclusion, variants in *CTNND1* can cause FEVR through the Wnt/cadherin axis.

## Graphical abstract

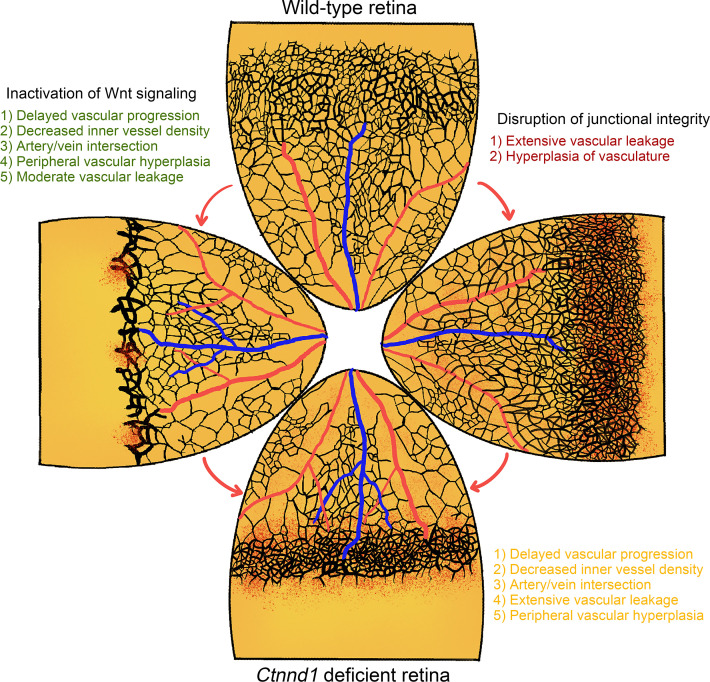

## Introduction

Vascular endothelial cells line the interior surface of blood vasculature, where they provide structural and functional barriers for the regulation of vascular permeability, inflammatory responses, and wound healing (1, 2). Blood vascular development is essential for embryogenesis and organogenesis, as well as for the maintenance of normal organ function. Consequently, defective vessel growth is associated with numerous congenital human diseases (3, 4). Familial exudative vitreoretinopathy (FEVR) is a severe genetic disorder in which incomplete peripheral retina vascularization causes secondary complications, including neovascularization, vitreous hemorrhage, retinal folds, and retinal detachments (5). A survey of early ocular examinations conducted in 64,632 Chinese newborns within 9 hospitals unraveled an incidence rate of FEVR of approximately 4.4‰ (6). Among the known genes involved in FEVR, components of the Wnt signaling pathway are highly prevalent, including *FZD4* (frizzled 4) (7), *LRP5* (low-density lipoprotein receptor-related protein 5) (8, 9), *TSPAN12* (tetraspanin 12) (10–12), *NDP* (norrin, previously known as Norrie disease pseudoglioma) (13), *LRP6* (low-density lipoprotein receptor-related protein 6) (14), and *CTNNB1* (catenin beta 1) (15–17). Additionally, variants identified in *ILK* (integrin-linked kinase) (18), *DLG1* (discs large MAGUK scaffold protein 1) (19), and *CTNNA1* (catenin alpha 1 or α-catenin) (20) were recently reported to cause FEVR through Wnt/β-catenin signaling.

The integrity of the blood vessel barrier is mainly regulated by the adherens junctions (AJs), indispensable cell-cell adhesion molecules at the plasma membrane where adjacent endothelial cells attach to each other with transmembrane linkers (21). The AJs are composed of cadherins and catenins, with the vascular endothelial–cadherin (VE-cadherin) (*CDH5*) serving as a central element that joins adjacent endothelial cells through calcium-dependent trans-interactions in the extracellular domain, whereas the cytoplasmic domain is associated with catenins including β-catenin (*CTNNB1*), γ-catenin (*JUP*), and p120-catenin (1, 22, 23). β-Catenin is also a key regulator of canonical Wnt signaling that binds to the carboxyl terminal domain of VE-cadherin and consequently associates VE-cadherin with the actin cytoskeleton through interactions with α-catenin (22, 24).

The armadillo protein p120 (encoded by the *CTNND1* gene) is a core stabilizer of the cadherin/catenin complex that binds to the juxtamembrane domain (JMD) of VE-cadherin in the endothelium (23, 25, 26). It has been reported that the p120 binding region of cadherins contains a dual-function motif with 9 amino acid residues (DEEGGGEMD) that alternately serve for endocytic signaling (first 3 amino acids DEE) or p120 binding (second 3 amino acids GGG); p120 binding inhibits cadherin endocytosis and promotes degradation by physically masking the endocytic signal at the JMD (23, 25, 26). In addition to regulating the function of cadherins, p120 has been reported to play key roles in the regulation of the Rho family of small GTPases (27), as well as Wnt signaling (28). Thus, p120 appears to function in cadherin-dependent or -independent ways. However, the distinct impacts of these different activities of p120 upon vascular development are poorly understood.

*CTNND1* is associated mostly with 2 human birth diseases, blepharocheilodontic (BCD) syndrome and nonsyndromic cleft palate (29–32). However, the link between *CTNND1* and FEVR has not yet been reported to our knowledge. In this study, we applied whole-exome sequencing (WES) on probands of 140 FEVR families and identified 3 candidate variants in the *CTNND1* gene. We further generated an inducible endothelial cell–specific (EC-specific) *Ctnnd1*-knockout mouse line to show that murine *Ctnnd1* is indispensable for postnatal retinal vascularization, which largely phenocopied FEVR-like vascular defects. Using in vivo and in vitro approaches combined with proteomic analysis and rescue assays, we concluded that p120 regulates Wnt signaling through direct stabilization of the cadherin/catenin complex and protection of β-catenin from glycogen synthase kinase 3 beta–ubiquitin–guided (Gsk3β-ubiquitin–guided) degradation. Variants in p120 caused disruption of the AJ integrity, as well as downregulation of Wnt signaling, by disturbing p120/VE-cadherin or p120/β-catenin interaction, which might ultimately cause FEVR. A double-heterozygous deletion study revealed distinct genetic interactions between p120 and β-catenin or α-catenin. These findings reveal a fundamental role for p120 in postnatal vascular development, which provides insights into the pathogenesis of FEVR and other vascular diseases.

## Results

### WES analysis of FEVR families identified variants in CTNND1.

**Figure 1:**
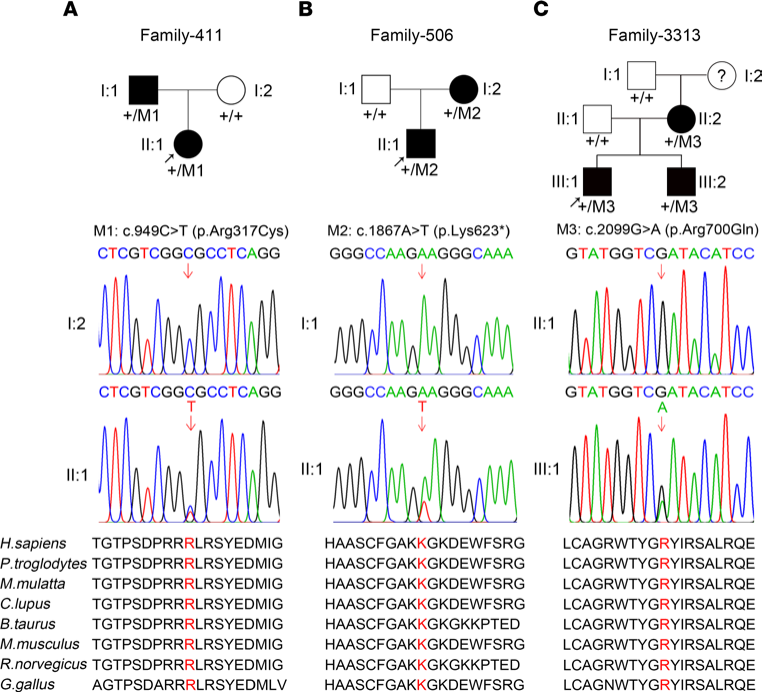
*CTNND1* variants in families with FEVR. (**A**–**C**) FEVR pedigrees and Sanger sequencing analysis showing presumed autosomal dominant inheritance of FEVR in Family-411 (**A**), Family-506 (**B**), and Family-3313 (**C**). Patients are denoted in black. Black arrows indicate the proband of each family. Red arrows indicate the changed nucleotides. Affected amino acids are denoted in red and are conserved among different species.

To identify potential candidate pathogenic variants for FEVR, we applied WES analysis to 140 FEVR-associated families without known causative variants. Presumed autosomal dominant variants identified by WES that had a frequency of less than 0.001 in the dbSNP151, Exome Variant Server, ExAC, 1000 Genomes Project, and gnomAD databases and were absent from 1000 sequenced controls were selected as candidate genes. Genes that met the following 3 standards were then selected (20): first, a candidate gene should have at least 3 variants in unrelated families; second, at least 1 “disruptive” variant (nonsense or frameshift) should be among the candidate variants; and third, the candidate gene should be related to Wnt signaling or angiogenesis. Thus, 3 potentially novel candidate variants in the *CTNND1* gene (NM_001085458) were identified in 3 Chinese families: a heterozygous variant c.949C>T (p.R317C) in Family-441, a heterozygous nonsense variant c.1867A>T (p.K623*) in Family-506, and a heterozygous missense variant c.2099G>A (p.R700Q) in Family-3313 (Figure 1, A–C). The WES results also showed no convincingly pathogenic variants in the known FEVR-associated genes in these probands. The pathogenicity of the variants was predicted to be deleterious by PolyPhen-2, Likelihood Ratio Test, and Mutation Taster software. The protein sequence alignment of human p120 with homologs from humans and other species indicated that all affected amino acids were highly conserved (Figure 1, A–C).

### Clinical findings.

**Figure 2:**
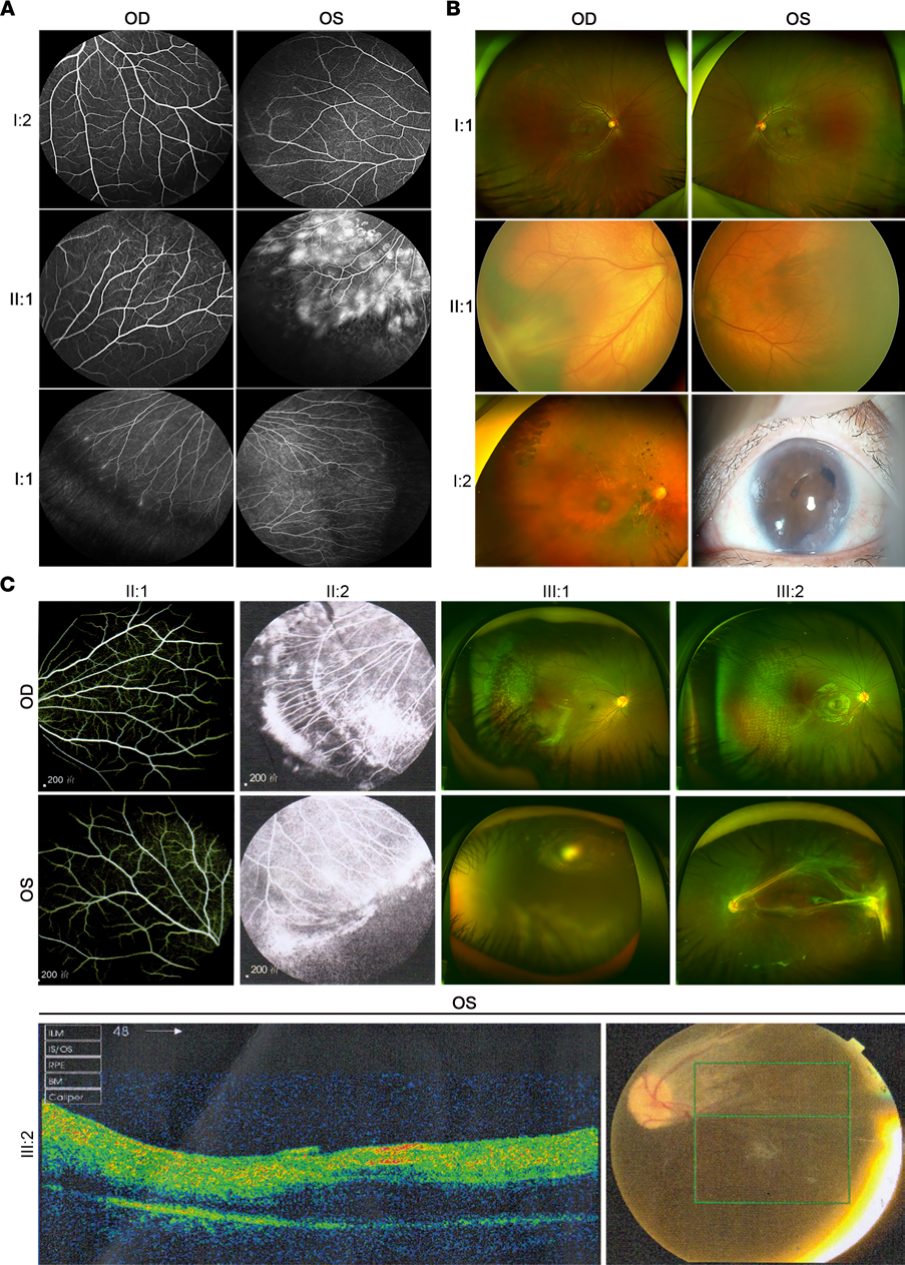
Clinical examination of the members in the FEVR-associated families. (**A**) Fundus fluorescein angiography (FFA) of Family-411 showing a retinal avascular area and peripheral vascular leakage of FEVR-affected proband (II:1); bilateral peripheral retinal avascular areas, straightened peripheral vessels, and increased vessel branching of the FEVR-affected father (I:1); and healthy vasculatures of the mother (I:2). (**B**) Optos examinations of Family-506 showing macular ectopia directing toward the temporal periphery in the right eye and a peripheral avascular area in the left eye of the FEVR-affected proband (II:1); macular ectopia and phthisis bulbi secondary to retinal detachment in the right eye of the FEVR-affected mother (I:2); and healthy vasculatures of the father (I:1). (**C**) FFA of the unaffected father (II:1) and FEVR-affected mother (II:2) showing bilateral peripheral avascular areas, exudation, and neovascularization. Optos examination of the FEVR-affected proband (III:1) showing peripheral avascular areas and severe retinal exudates in the right eye and band-shaped keratopathy in the left eye. Optos, optical coherence tomography (OCT), and fundus examination of his younger brother (III:2) showing peripheral avascular areas and exudation in the right eye, and retinal detachment in the left eye. Green box in the fundus photograph indicates scanned areas in the OCT examination. OD, oculus dexter; OS, oculus sinister.

For the heterozygous c.949C>T (p.R317C) variant in Family-441, the proband was diagnosed with FEVR at age 6, manifesting severe retinal exudates with a characteristic peripheral avascular retina in the left eye (Figure 2A). His father was heterozygous and manifested a bilateral peripheral retinal avascular area, straightened peripheral vessels, and increased vessel branching, while his mother showed a normal fundus without this variant (Figure 2A).

The second heterozygous variant, c.1867A>T (p.K623*), was found in a 1-month-old boy with macular ectopia directed toward the temporal periphery in the right eye and a peripheral avascular area in the left eye (Figure 2B). The proband also manifested with features of BCD syndrome phenotypes, including syndactyly, and cleft lip/palate (CLP) (Figure 2B). His affected mother was heterozygous and manifested macular ectopia and phthisis bulbi secondary to retinal detachment in the right eye, while his father manifested normal visual acuity and fundus without this variant (Figure 2B). The mother of the proband also manifested distinct BCD syndrome phenotypes (syndactyly without CLP). The K623* was a nonsense variant that was predicted to result in premature transcription termination. We thus speculated that the K623* variant might be a major pathogenic variant in this family.

The third heterozygous variant, c.2099G>A (p.R700Q), was found in an 8-year-old boy with peripheral avascularization and severe retinal exudates in the right eye, whereas the vasculature of his left eye was undetectable due to band keratopathy (Figure 2C). The younger brother of the proband was heterozygous for the variant, manifesting a similar phenotype in the right eye and retinal detachment in the left (Figure 2C). Their mother was heterozygous for this variant, manifesting bilateral peripheral avascularization, exudation, and neovascularization (Figure 2C). The father and maternal grandfather were normal without this variant.

Thus, these variants in *CTNND1* cosegregated with the disease phenotype in their families (Figure 1); pathogenesis of these variants that cause FEVR was further investigated by in vivo and in vitro experiments.

### Loss of Ctnnd1 in ECs results in FEVR-like phenotypes.

Given that depletion of mouse endothelial p120 using a conditional knockout system promoted by *Tie2-Cre* results in embryonic lethality (33), we generated an inducible EC-specific *Ctnnd1*-knockout mouse model by breeding tamoxifen-induced *Pdgfb-iCreER* transgenic mice with mice harboring the *Ctnnd1* *loxP*-flanked allele to evaluate the function of p120 in the retinal vascularization of postnatal mice (Supplemental Figure 1, A–C; supplemental material available online with this article; https://doi.org/10.1172/jci.insight.158428DS1). The *Ctnnd1^fl/fl^* *Pdgfb-iCre-ER* (*Ctnnd1^iECKO/iECKO^*) mice, *Ctnnd1^fl/+^* *Pdgfb-iCre-ER* (*Ctnnd1^iECKO/+^*) mice, and their control littermates (*Ctnnd1^fl/fl^* mice or *Ctnndl^+/+^* *Pdgfb-iCre-ER* mice) were treated with tamoxifen for 3 consecutive days, from postnatal day 1 (P1) to P3 (Supplemental Figure 1C). Western blot and real-time quantitative PCR (RT-qPCR) analysis revealed a more than 80% decrease in p120 protein and mRNA from P25 *Ctnnd1^iECKO/iECKO^* mouse lungs, indicating reliable inactivation of *Ctnnd1* in the vascular ECs (Supplemental Figure 1, D–F).

**Figure 3:**
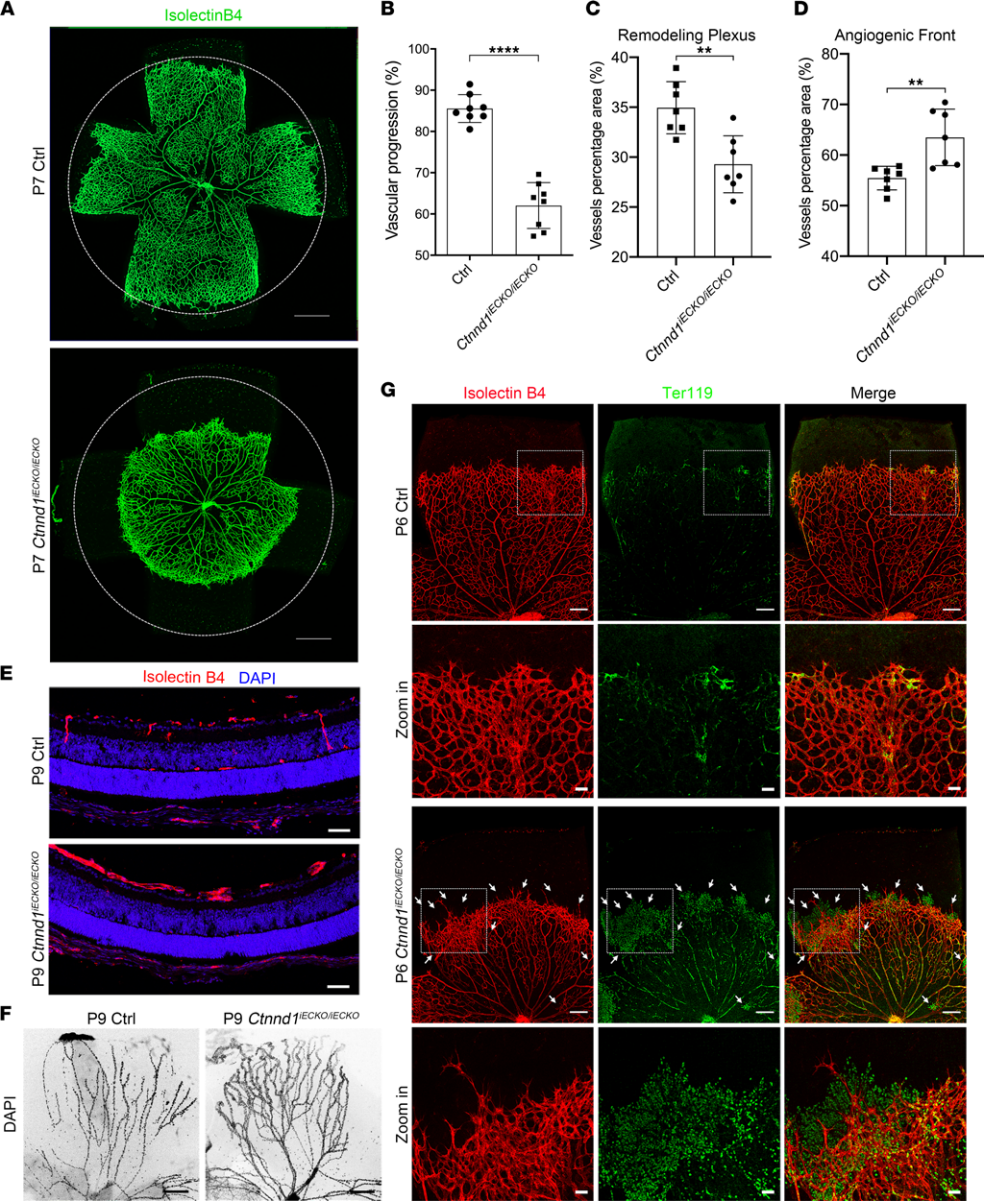
Loss of *Ctnnd1* in mouse ECs impairs angiogenesis and hyaloid regression. (**A**) Representative overview of P7 control (Ctrl) and *Ctnnd1^iECKO/iECKO^* mouse retinas labeled with isolectin B4 (IB4). The circles indicate vessel outgrowth in the Ctrl group. Scale bars, 500 μm. (**B**–**D**) Quantification of vascular progression (**B**), vascular density in the remodeling plexus (**C**), and angiogenic front (**D**) of P6 Ctrl and *Ctnnd1^iECKO/iECKO^* mouse retinas. Error bars, standard deviations (SDs). Student’s *t* test (*n* ≥ 7), **P < 0.01, ****P < 0.0001. (**E**) Representative immunofluorescence images of cross sections of retinas from P9 Ctrl and *Ctnnd1^iECKO/iECKO^* mice costained with IB4 (red) and DAPI (blue). Scale bars, 20 μm. (**F**) Representative immunofluorescence images of hyaloid vessels from P9 Ctrl and *Ctnnd1^iECKO/iECKO^* mice. Scale bars, 200 μm. (**G**) Representative overview and high-magnification immunofluorescence images of P6 Ctrl and *Ctnnd1^iECKO/iECKO^* mouse retinas labeled with IB4 (red) and Ter119 (green). Dotted boxes indicate magnified areas. White arrows indicate abnormal leakage of erythrocytes. Scale bars, 200 μm and 50 μm. Experiments were performed at least 3 times independently.

The development of mouse retinal vasculature initiates from the optic nerve; the tip cells at the leading edge (hereafter termed the angiogenic front) extend numerous filopodia and radially sprout to form the superficial vasculature (10, 34). The primary vessel plexus behind the tip cells (hereafter termed the remodeling plexus) subsequently undergoes pruning to form a hierarchically mature vasculature (10, 34). To evaluate whether deletion of p120 could cause FEVR-like phenotypes, we first applied immunofluorescence analysis on the P7 flat-mounted retinas and observed compromised radial outgrowth of retinal vessels upon deletion of endothelial *Ctnnd1* in mice (Figure 3, A and B). Interestingly, the vascular density of the *Ctnnd1^iECKO/iECKO^* mouse retinas was reduced in the remodeling plexus (Figure 3, A and C), whereas it was elevated in the angiogenic front (Figure 3, A and D), demonstrating vascular hyperplasia at the peripheral retina (10, 34). After the initial outgrowth of the superficial layers from P1 to P7, the retinal vessels sprout perpendicularly into the deep layers and ramify in the outer plexiform layers (OPLs) and inner plexiform layers, leading to the formation of a 3-layered retinal vascular architecture (35, 36). At P9, vertical sprouts and OPL capillaries appeared in the control mice, whereas both were absent in the *Ctnnd1^iECKO/iECKO^* mice (Figure 3E).

During postnatal vascular development, the hyaloid vessels reach maximal development at approximately P3 and start to regress in the second week (34, 36). Delayed hyaloid regression has previously been reported in several FEVR-associated mouse models (10, 20, 34, 36, 37). As predicted, we observed retarded regression of hyaloid vessels in the P9 *Ctnnd1^iECKO/iECKO^* mouse retinas, as compared with their littermate controls (Figure 3F). Given that regression of hyaloid vessels begins from P4 and finishes by P21 in mice (38), we further tested whether hyaloid vessel is persistent in P21 *Ctnnd1^iECKO/iECKO^* mice. Hematoxylin and eosin staining of paraffin-embedded ocular sections showed remanent hyaloid vessels in P21 *Ctnnd1^iECKO/iECKO^* mice, which were absent in wild-type mice (Supplemental Figure 2A). Additionally, we did not observe structural abnormalities in the eyes of *Ctnnd1^iECKO/iECKO^* mice, as compared with those of littermate controls (Figure 3E and Supplemental Figure 2A).

The integrity of the inner blood-retinal barrier (BRB) is critical for selective vascular permeability, and disruption of BRB integrity might lead to severe leakage, which is a typical phenotype of FEVR (10, 13, 20, 34, 36, 39, 40). In our study, Ter119-labeled erythrocytes were confined to the vasculature of P6 control retinas (Figure 3G). However, a large abundance of erythrocytes was detected outside the vessel lumen in the retinas of P6 *Ctnnd1^iECKO/iECKO^* mice (Figure 3G), indicating the disruption of BRB upon depletion of *Ctnnd1*. Biocytin-TMR is an 869 Da molecule that ordinarily crosses the BRB when paracellular junctional integrity is disrupted (41). We therefore performed injection of biocytin-TMR to detect the effect of *Ctnnd1* depletion on BRB integrity. As expected, loss of p120 in mouse ECs resulted in enhanced vascular permeability for biocytin-TMR at P6 (Supplemental Figure 2B), indicative of disrupted BRB integrity. p120 was previously reported to facilitate the anchoring of VE-cadherin to the plasma membrane (42). To evaluate the localization of VE-cadherin in the absence of p120, we stained the P6 retina flat mounts with antibodies against VE-cadherin. VE-cadherin outlined the retinal vasculatures of the P6 control mice, while it was abnormally accumulated and diffusely distributed with a less clear vessel boundary distribution in the retinas of *Ctnnd1^iECKO/iECKO^* mice, indicating disruption of the AJ continuity (Supplemental Figure 2C). Meanwhile, the ultrastructure of P9 ECs under transmission electron microscopy showed a discontinuous EC-EC junction in the absence of p120 (Supplemental Figure 2D), confirming disruption of the endothelial junctions.

**Figure 4:**
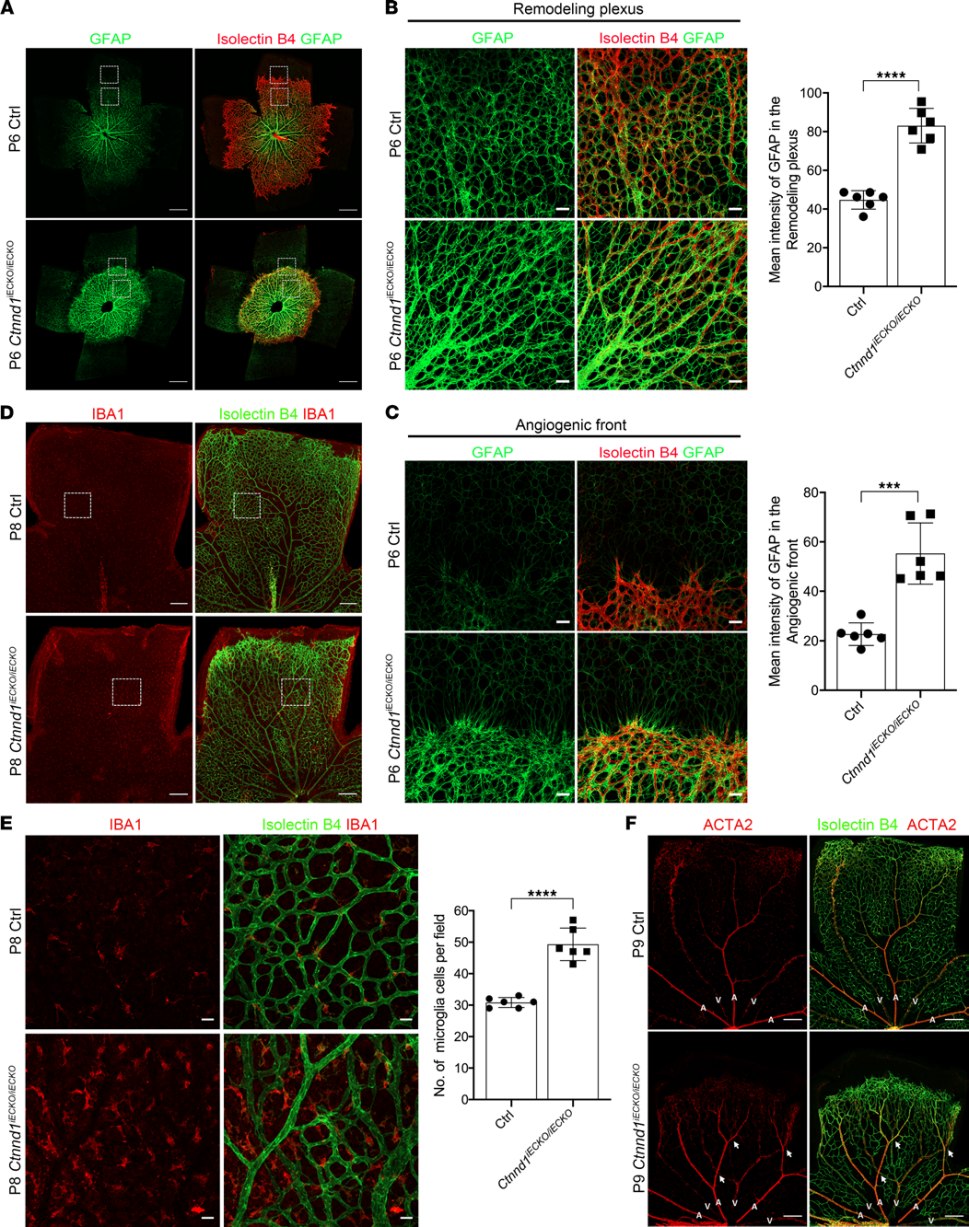
Loss of *Ctnnd1* in mouse ECs leads to artery-vein intersection and reactive gliosis in the mouse retinas. (**A**) Representative overview of P6 Ctrl and *Ctnnd1^iECKO/iECKO^* mouse retinas labeled with IB4 (red) and GFAP (green). Dotted boxes indicate magnified areas shown in **B** and **C**. Scale bars, 500 μm. (**B** and **C**) High-magnification immunofluorescence images and quantification of mean GFAP intensity in the remodeling plexus (**B**) and angiogenic front (**C**) of P6 Ctrl and *Ctnnd1^iECKO/iECKO^* mouse retinas. Error bars, SDs. Student’s *t* test (*n* = 6), ***P < 0.001, ****P < 0.0001. Scale bars, 20 μm. (**D**) Representative immunofluorescence images of P8 Ctrl and *Ctnnd1^iECKO/iECKO^* mouse retinas labeled with IB4 (green) and IBA1 (red). Dotted boxes indicate magnified areas. Scale bars, 200 μm. (**E**) High-magnification immunofluorescence images and quantification of the number of IBA1-expressing microglia in P6 Ctrl and *Ctnnd1^iECKO/iECKO^* mouse retinas. Error bars, SDs. Student’s *t* test (*n* = 6), ****P < 0.0001. Scale bars, 20 μm. (**F**) Representative immunofluorescence images of P9 Ctrl and *Ctnnd1^iECKO/iECKO^* mouse retinas labeled with IB4 (green) and ACTA2 (red). The white arrows indicate abnormal crossings of major arteries and veins. Scale bars, 200 μm. A, artery; V, vein. Experiments were performed at least 3 times independently.

Since an increased level of glial fibrillary acid protein (GFAP) has previously been reported to indicate active astrocytes in *Fzd4*, *Norrin*, *Tspan12*, or *Ctnna1* mutant mouse retinas (10, 20, 34, 37), we stained GFAP in the P6 retinas of *Ctnnd1^iECKO/iECKO^* mice and control littermates to evaluate the activation of astrocytes. The results showed prominently higher GFAP intensities in both the angiogenic front and remodeling plexus of *Ctnnd1^iECKO/iECKO^* mouse retinas compared with littermate controls, indicating overactivation and gliosis of astrocytes (Figure 4, A and B). Astrocytes and microglia are resident immune cells that act as primary defenses in the retina. Reactive astrogliosis and microgliosis are involved in immune responses (43, 44). We thus performed ionized calcium-binding adapter molecule 1 (IBA1) staining to detect whether microglia were reactive in the absence of EC-derived p120. As expected, we observed abnormal proliferation of amoeboid-shaped microglia with enlarged cell bodies in the retinas of P8 *Ctnnd1^iECKO/iECKO^* mice (Figure 4, C and D), which are markers of reactive microglia (44). These results indicated reactive gliosis in the *Ctnnd1^iECKO/iECKO^* mouse retinas.

A mature vasculature can be divided into arteries, veins, and capillaries. In P9 wild-type retinas, the major arteries and veins were segregated into alternating and radially arrayed territories (Figure 4E); however, several crossings between arteries and veins were observed in the P9 *Ctnnd1^iECKO/iECKO^* mouse retinas (Figure 4F), which closely resembles the phenotype observed in *Ndp^KO^* retinas (36).

### Loss of Ctnnd1 causes abnormal VEGF expression, cell proliferation, and vessel pruning.

To further explore the mechanism of defective vascular development in *Ctnnd1^iECKO/iECKO^* mouse retinas, we first asked whether the dense appearance of the distal vasculature resulted from filopodia-extending tip cell hyperproliferation. The results showed that tip cells at the leading edge were more abundant in the P6 *Ctnnd1^iECKO/iECKO^* retinas than in those of the littermate controls (Supplemental Figure 3, A and B), suggesting abnormal proliferation of *Ctnnd1*-null ECs at the peripheral area of the retinal vasculature. We further applied EdU incorporation assay to confirm whether mitotic cell proliferation of the peripheral ECs increased. Consistently, the number of relative EdU^+^ ECs drastically increased at the angiogenic front of the P6 *Ctnnd1^iECKO/iECKO^* mouse retinas (Supplemental Figure 3, C and D).

Normally, astrocytes prominently secrete VEGF in the angiogenic front and form a gradient in the avascular areas ahead of the vessel plexus, regulating tip cell proliferation and extension during retinal vascular development (45). To explore the mechanism of hyperplasia and delayed radial outgrowth in p120-defective retinal vessels, we examined the abundance of Vegfa in the P6 *Ctnnd1^iECKO/iECKO^* and control retinas. Fluorescence immunostaining of flat mounts showed strong upregulation of Vegfa expression in both the angiogenic front and the avascular areas upon *Ctnnd1* depletion (Supplemental Figure 4, A–C), presumably in response to erythrocyte leakage and retinal hypoxia. However, the Vegfa level in the remodeling plexus was comparable between the control and *Ctnnd1^iECKO/iECKO^* retinas (Supplemental Figure 4, B and C).

Collagen IV is a component of matrix deposits that regressing vascular ECs leave (46). Collagen IV^+^ and isolectin B4^–^ matrix sleeves, which indicate vessel pruning, were highly abundant in P6 *Ctnnd1^iECKO/iECKO^* mouse retinas (Supplemental Figure 4D), indicating that loss of p120 function might lead to abnormal vessel pruning. This may be responsible for decreased vessel density in the remodeling plexus (Figure 3, A and C).

### Depletion of CTNND1 in human retinal microvascular endothelial cells inhibits the cadherin and Wnt signaling pathways.

**Figure 5:**
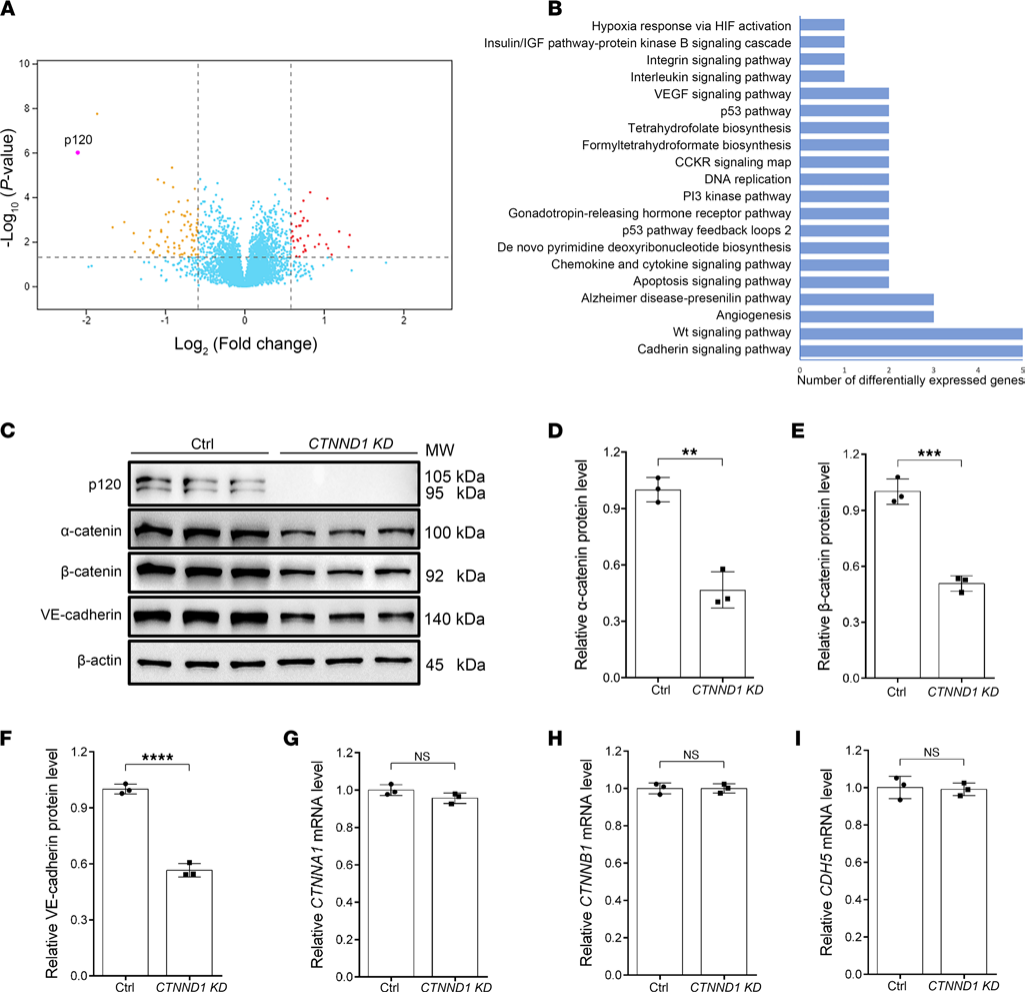
p120 mainly participates in cadherin and Wnt signaling in HRECs. (**A**) Volcano plot of differentially expressed genes detected by unbiased proteomics in the *CTNND1*-KD HRECs compared to Ctrl HRECs. The *P* values (–log_10_ transformed) are displayed as a function of the log_2_-transformed fold changes. The dashed lines indicate the threshold of *P* < 0.05 and fold change > 1.5. The sorted downregulated and upregulated genes are shown as yellow and red dots, respectively; genes without significant alteration are shown as blue dots. (**B**) Pathway classification of differentially expressed proteins using the PANTHER classification system online, showing remarkable enrichment for cadherin and Wnt signaling pathways. (**C**) Western blot analysis of protein levels of cadherin/catenin components (p120, α-catenin, β-catenin, and VE-cadherin) in Ctrl and *CTNND1*-KD HRECs. (**D**–**F**) Quantification of relative α-catenin, β-catenin, and VE-cadherin protein levels in Ctrl and *CTNND1*-KD HRECs. Error bars, SDs. Student’s *t* test (*n* = 3), **P < 0.01, ***P < 0.001, ****P < 0.0001. (**G**–**I**) Quantification of relative *CTNNA1*, *CTNNB1*, and *CDH5* mRNA levels detected by real-time quantitative PCR (RT-qPCR) in Ctrl and *CTNND1*-KD HRECs. Error bars, SDs. Student’s *t* test (*n* = 3). Experiments were performed at least 3 times independently.

**Figure 6:**
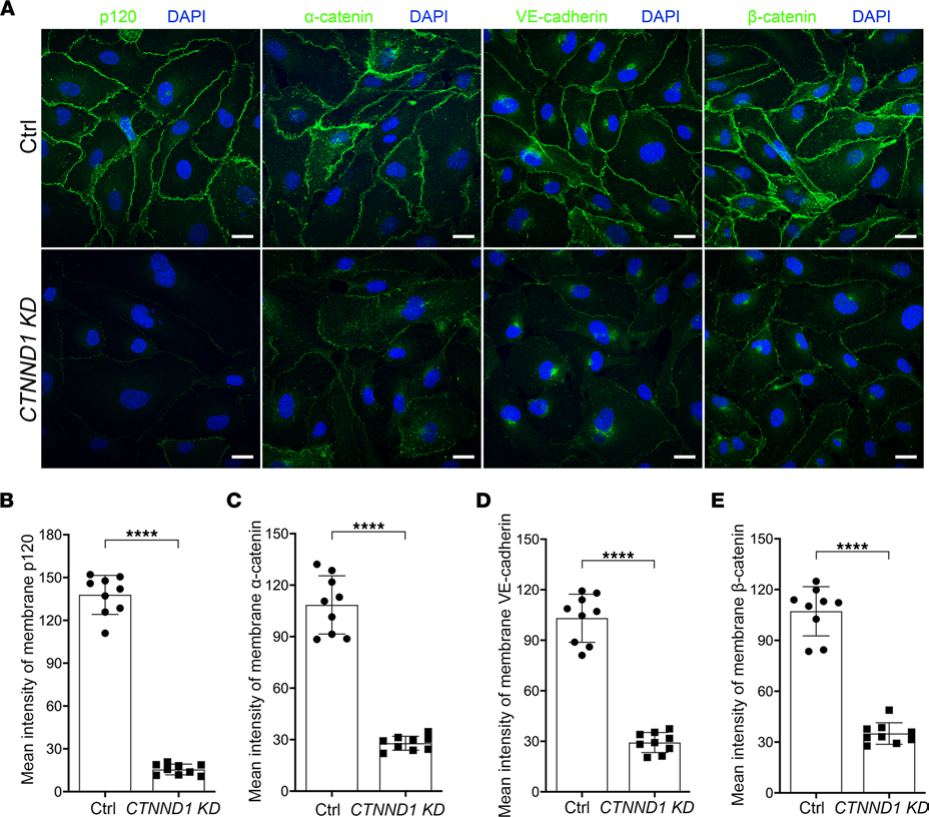
Depletion of *CTNND1* in HRECs results in reduced junctional integrity. (**A**) Representative immunofluorescence images of Ctrl and *CTNND1*-KD HRECs labeled with DAPI (blue) and p120, α-catenin, VE-cadherin, or β-catenin (green), respectively. Scale bars, 20 μm. (**B**–**E**) Quantification of mean intensity of membrane-localized p120, α-catenin, VE-cadherin, and β-catenin in Ctrl and *CTNND1*-KD HRECs. Error bars, SDs. Student’s *t* test (*n* = 9), ****P < 0.0001. Experiments were performed at least 3 times independently.

To explore the molecular mechanism through which p120 regulates EC function, we applied lentivirus-directed *shRNA* knockdown of *CTNND1* to primary human retinal microvascular endothelial cells (HRECs). An unbiased proteomic analysis was performed on the control and *CTNND1*-knockdown HRECs. A large number of differentially expressed proteins were detected, as shown in the volcano plot, and the abundance of the p120 protein was confirmed to be substantially decreased (Figure 5A and Supplemental Table 1). Of those involved, the cadherin and Wnt signaling pathways were the most affected upon knockdown of *CTNND1* (Figure 5B). We first focused on the cadherin signaling pathway and applied Western blot analysis to the HREC lysates. In the absence of p120, the protein levels of α-catenin, β-catenin, and VE-cadherin were considerably downregulated (Figure 5, C–F), whereas RT-qPCR analysis revealed unchanged mRNA levels for these genes (Figure 5, G–I), indicating that p120 regulates the expression of cadherin/catenin components at the protein level rather than at the mRNA level. We further applied immunofluorescence staining to evaluate the AJ integrity of p120-defective HRECs. The intensities of membrane-localized cadherin/catenin complex proteins in *CTNND1*-depleted HRECs were found to be remarkably decreased compared with those in control HRECs (Figure 6, A–E), which might lead to vessel leakage in vivo. These results suggested that p120 might play a pivotal role in the regulation of AJ integrity and protect cadherin/catenin components from degradation.

We recently reported that inactivation of *CTNNA1* (α-catenin) in ECs results in reduced membrane localization of the cadherin/catenin complex (20). However, to our knowledge it has not been determined whether depletion of α-catenin affects the total protein levels of cadherin/catenin components. Interestingly, Western blot analysis of HRECs transfected with control or *CTNNA1* *shRNA* exhibited identical total protein levels of other cadherin/catenin components (Supplemental Figure 5), further indicating that p120 might be a specific regulator protecting the cadherin/catenin complex from degradation.

**Figure 7:**
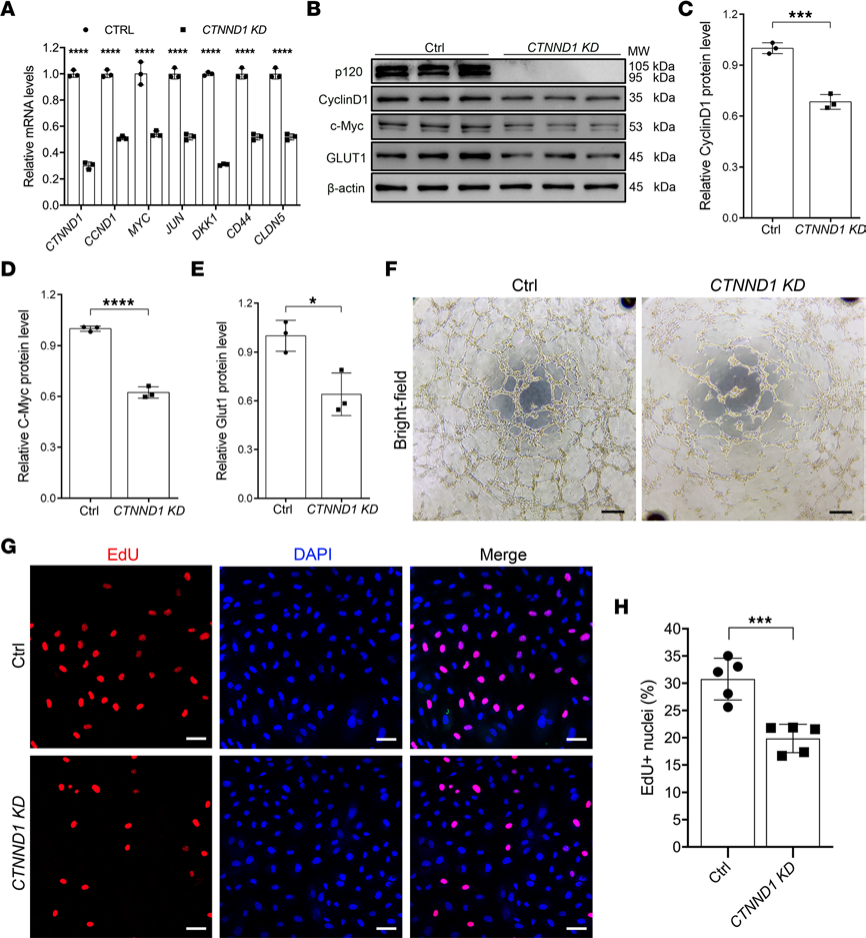
Depletion of *CTNND1* in HRECs inhibits in vitro EC angiogenesis and cell proliferation partially through inactivation of Wnt signaling activity. (**A**) Quantification of relative *CTNND1*, *CCND1*, *MYC*, *JUN*, *DKK1*, *CD44*, and *CLDN5* mRNA levels detected by RT-qPCR in Ctrl and *CTNND1*-KD HRECs. Error bars, SDs. Student’s *t* test (*n* = 3), ****P < 0.0001. (**B**) Western blot analysis of protein levels of Wnt signaling targets (p120, CyclinD1, c-Myc, and GLUT1) in Ctrl and *CTNND1*-KD HRECs. (**C**–**E**) Quantification of relative CyclinD1, c-Myc, and GLUT1 protein levels in Ctrl and *CTNND1*-KD HRECs. Error bars, SDs. Student’s *t* test (*n* = 3), *P < 0.05, ***P < 0.001, ****P < 0.0001. (**F**) Representative bright-field images of in vitro tube formation in Ctrl and *CTNND1*-KD HRECs. Scale bars, 25 μm. (**G**) Representative immunofluorescence images of Ctrl and *CTNND1*-KD HRECs labeled with EdU (red) and DAPI (blue). Scale bars, 50 μm. (**H**) Quantification of the percentage of EdU^+^ nuclei in Ctrl and *CTNND1*-KD HRECs. Error bars, SDs. Student’s *t* test (*n* = 5), ***P < 0.001. Experiments were performed at least 3 times independently.

Notably, in addition to functioning in cadherin signaling, β-catenin is more widely known as a core component of the Wnt signaling pathway. We next asked whether the Wnt signaling pathway was disturbed in *CTNND1*-depleted HRECs. The mRNA levels of several Wnt-targeted genes, including *CyclinD1* (*CCND1*), *c-Myc* (*MYC*), *c-Jun* (*JUN*), *DKK1*, *CD44*, and *claudin5* (*CLDN5*), were significantly reduced in the absence of p120 (Figure 7A). Additionally, we observed an appreciable reduction of the CyclinD1, c-Myc, and glucose transporter type 1 (GLUT1) proteins in *CTNND1*-depleted HRECs (Figure 7, B–E).

Since defective Wnt signaling was previously reported to result in compromised in vitro vascularization (46–48), we applied a Matrigel tube formation assay to investigate in vitro vascularization in *CTNND1*-depleted HRECs. As expected, we observed defective in vitro tube formation upon deletion of *CTNND1* in the HRECs (Figure 7F). Given that Wnt signaling regulates vascular cell proliferation (36), we unsurprisingly detected decreased mitotic cell proliferation in *CTNND1*-depleted HRECs (Figure 7, G and H), which is consistent with the consequence of defective Wnt signaling. These results confirmed the compromised activity of the endogenous Wnt/β-catenin signaling pathway in the absence of p120, which might cause defective proliferation and vascularization of HRECs.

We noticed a discrepancy between the compromised HREC proliferation and the increased EC proliferation of *Ctnnd1^iECKO/iECKO^* in the angiogenic front. Since levels of Vegfa, a master regulator of EC proliferation, were increased in the angiogenic front and avascular areas of the *Ctnnd1^iECKO/iECKO^* mouse retinas (Supplemental Figure 4, A–C), we hypothesized that excessive Vegfa secreted by astrocytes might be attributable to hyperplasia of the peripheral retinal vasculature. Therefore, we applied recombinant VEGF-165 to test VEGF signaling responses in control and *CTNND1*-depleted HRECs. Western blot analysis revealed that treatment with VEGF-165 led to elevated phosphorylation of VEGFR2 and ERK1/2 in both control and *CTNND1*-depleted HRECs (Supplemental Figure 6). Intriguingly, in the presence of VEGF-165, the phosphorylation levels of VEGFR2 and ERK1/2 were higher in the *CTNND1*-depleted HRECs than that in the control (Supplemental Figure 6), suggesting that HRECs were more sensitive to VEGF-165 upon *CTNND1* depletion. Additionally, we also investigated the effect of VEGF-165 on Wnt signaling activity. However, we did not observe alterations of β-catenin and CyclinD1 protein levels upon VEGF-165 treatment, neither in control nor in *CTNND1*-depleted HRECs (Supplemental Figure 6).

**Figure 8:**
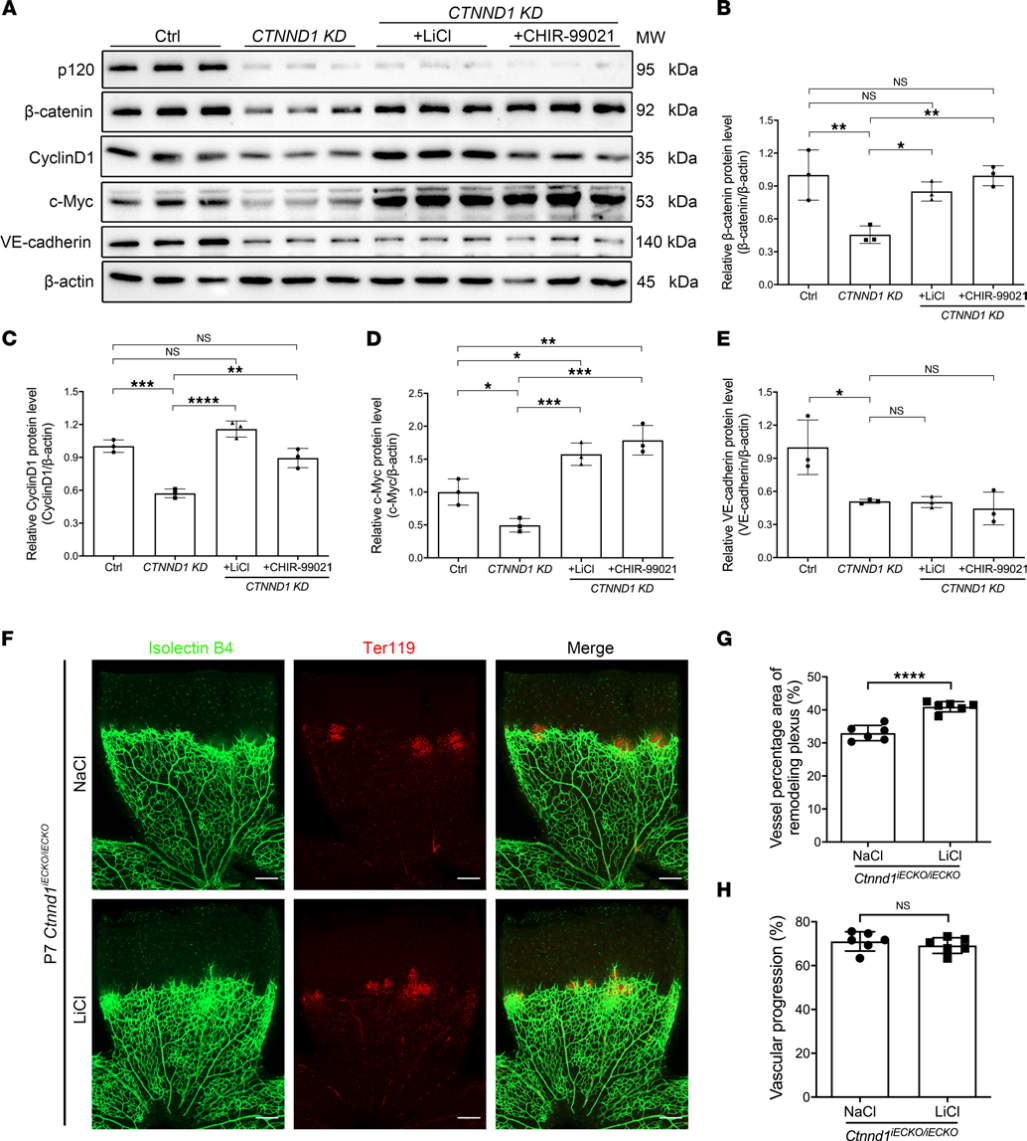
Treatment with LiCl or CHIR-99021 corrects the Wnt signaling activity in *CTNND1*-KD HRECs and the vascular density of *Ctnnd1^iECKO/iECKO^* mouse retinas. (**A**) Western blot analysis of protein levels of β-catenin, VE-cadherin, c-Myc, and CyclinD1 in Ctrl and *CTNND1*-KD HRECs treated with LiCl, CHIR-99021, or vehicle. (**B**–**E**) Quantification of relative protein levels of β-catenin, VE-cadherin, c-Myc, and CyclinD1 in Ctrl and *CTNND1*-KD HRECs treated with LiCl, CHIR-99021, or vehicle. Error bars, SDs. The *P* values are from multiple comparisons in 1-way ANOVA with Dunnett’s or Tukey’s multiple comparisons test (*n* = 3). *P < 0.05, **P < 0.01, ***P < 0.001, ****P < 0.0001. (**F**) Representative immunofluorescence images of IB4-labeled (green) and Ter119-labeled (red) retina flat mounts from P7 *Ctnnd1^iECKO/iECKO^* mice treated with LiCl or NaCl. Scale bars, 200 μm. (**G** and **H**) Quantification of vascular density of remodeling plexus (**G**) and vascular progression (**H**) in P7 *Ctnnd1^iECKO/iECKO^* mouse retinas treated with LiCl or NaCl. Error bars, SDs. Student’s *t* test (*n* = 6), ****P < 0.0001. Experiments were performed at least 3 times independently.

It has been widely established that Gsk3β regulates phosphorylation of β-catenin at the Ser33, Ser37, and Thr41 residues, providing a docking site for E3 ubiquitin ligase β-TrCP, which mediates ubiquitination and subsequent proteasomal degradation of β-catenin (49, 50). Thus, we asked whether p120 protects β-catenin from degradation by inhibiting this Gsk3β-mediated process. We applied treatment with LiCl or CHIR-99021, inhibitors of Gsk3β, to restore β-catenin signaling in p120-deficient HRECs. Western blot analysis showed that LiCl or CHIR-99021 considerably restored the endogenous protein levels of β-catenin, as well as downstream targets CyclinD1 and c-Myc (Figure 8, A–E). Not surprisingly, both LiCl and CHIR-99021 failed to restore the protein levels of VE-cadherin (Figure 8, A and E). Immunofluorescence staining also showed that treatment with LiCl or CHIR-99021 partially restored the levels of membrane- and nucleus-localized β-catenin in p120-deficient HRECs (Supplemental Figure 7, A and B). Additionally, treatment with LiCl or CHIR-99021 remarkably promoted cell proliferation in *CTNND1-*depleted HRECs (Supplemental Figure 7, C and D). These results suggested that p120 protects β-catenin from degradation and that loss of p120 might impair the proliferation of ECs through inactivation of Wnt signaling, which could be corrected by inhibition of Gsk3β-ubiquitin-guided β-catenin degradation.

To explore whether *Ctnnd1* depletion affects Wnt signaling in vivo, a survey of several markers of the Wnt-regulated genes in the retinal vasculature was conducted to show a decrease of lymphoid enhancer binding factor 1 (LEF1), a transcriptional mediator of Norrin/β-catenin signaling (Supplemental Figure 8, A and B) (18), and an increase of plasmalemma vesicle-associated protein, an endothelial fenestrae marker (36, 48, 51) (Supplemental Figure 8, C and D), in the P6 *Ctnnd1^iECKO/iECKO^* mouse retinal vasculature. In addition, Western blot analysis of the P25 mouse lung lysates revealed that knockout of *Ctnnd1* in mouse ECs caused compromised cadherin and Wnt signaling (Supplemental Figure 8, E–K). These results supported the notion of defective Wnt signaling in the retinal vascular ECs of *Ctnnd1^iECKO/iECKO^* mice.

Moreover, given that LiCl has previously been applied to normalizing ocular vessels in *Lrp5*^–/–^ and heterozygous EC-specific *Ctnnb1*-knockout mice (17, 38), we applied intraperitoneal injection of LiCl (10 mg/kg) to *Ctnnd1^iECKO/iECKO^* mice to investigate whether overactivation of β-catenin signaling could rescue defective retinal angiogenesis. Interestingly, LiCl treatment considerably restored vascular density in the remodeling plexus of P7 *Ctnnd1^iECKO/iECKO^* mouse retinas (Figure 8, F and G). However, LiCl failed to promote vascular progression and prevent leakage of erythrocytes in the *Ctnnd1^iECKO/iECKO^* mouse retinal vasculature (Figure 8, F and H).

### CTNND1 variants disrupt the cadherin/catenin complex and inactivate Wnt signaling activity.

**Figure 9:**
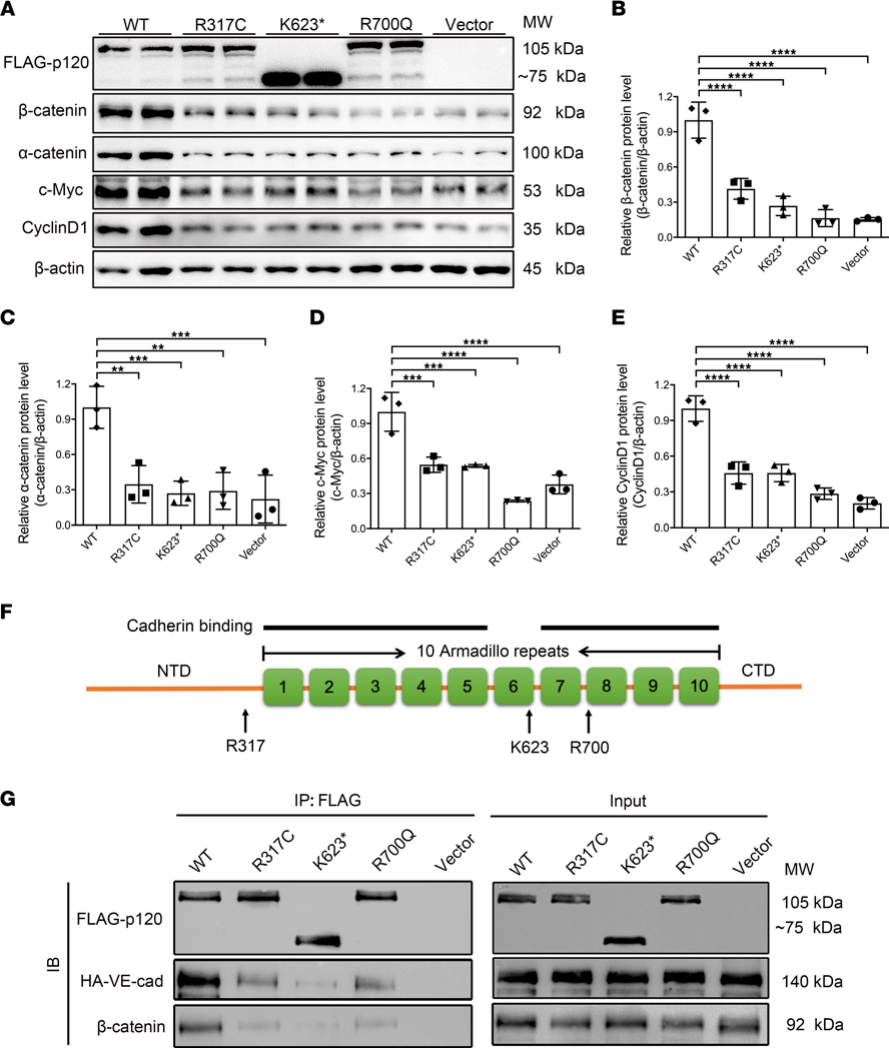
Functional consequences of *CTNND1* variant alleles. (**A**) Western blot analysis of protein levels of overexpressed FLAG-p120 and endogenous β-catenin, α-catenin, c-Myc, and CyclinD1 in HEK293T cells transfected with wild-type (WT), variant, or vector plasmids. (**B**–**E**) Quantification of relative protein levels of endogenous β-catenin, α-catenin, c-Myc, and CyclinD1 in HEK293T cells transfected with WT, variant, or vector plasmids. The *P* values are from multiple comparisons in 1-way ANOVA with Dunnett’s multiple comparisons test (*n* = 3); **P < 0.01, ***P < 0.001, ****P < 0.0001. (**F**) Schematic diagram of p120 protein. p120 consists of 10 armadillo repeat (ARM) domains flanked by N-terminal (NTD) and C-terminal (CTD) domains. The potential binding surfaces for cadherins are indicated with black lines. The locations of mutated residues are indicated with black arrows. (**G**) Western blot analysis of FLAG-p120 (WT, variant, or vector) co-immunoprecipitated with HA-VE-cadherin (HA-VE-cad) or endogenous β-catenin. An empty vector was used as negative control. Experiments were performed at least 3 times independently.

To confirm the effects of the *CTNND1* variants R317C, K623*, and R700Q on the biological functions of p120, we applied site-directed mutagenesis to introduce these variants into *CTNND1* expression plasmids, which are resistant to *shRNA* targeting without changing the amino acid sequence. Wild-type *CTNND1*, variant *CTNND1*, or vector plasmids were then transfected into *CTNND1*-knockdown HEK293T cells to evaluate the effects of variants on the function of the p120 protein. As predicted, Western blot analysis showed truncation of the K623* variant without affecting protein levels, while the R317C and R700Q variants produced identical bands to the wild-type *CTNND1* expression plasmid (Figure 9, A–E). Notably, overexpression of wild-type protein remarkably increased the endogenous levels of β-catenin, which further triggered activation of Wnt-targeted genes, including CyclinD1 and c-Myc (Figure 9, A–E). However, the R317C, K623*, and R700Q variants failed to restore expression levels of β-catenin, α-catenin, and Wnt-targeted genes, indicating that these variants might disrupt the normal function of p120 (Figure 9, A–E).

The currently available structural and mutagenesis analyses exhibited direct interaction between p120 and E-cadherin via broad cadherin binding interfaces in the p120 armadillo repeat (ARM) domains (1–5 and 7–10 ARM domains) (52, 53). The R700 residue is located in the eighth ARM domain of p120 (Figure 9F), and substitution of Arg with Cys might disrupt potential p120/cadherin binding sites. The Lys623* variant was proved to result in a truncated protein that lacked the 7–10 ARM domains and the C-terminal domain (Figure 9F), which is likelier than the R700Q variant to cause disrupted p120/cadherin binding surfaces. The R317 residue is located in the N-terminal domain, which is structurally adjacent to the first ARM domain (Figure 9F). However, since R317 was not included in the structure currently available, the exact role of this residue in the binding of p120 and cadherin is unknown. To investigate the mechanism by which these variants affect the cadherin/catenin complex and Wnt signaling activity, we tested whether these variants disrupt interactions between p120 and VE-cadherin or β-catenin using co-immunoprecipitation analysis. The results revealed that wild-type p120 strongly interacted with overexpressed VE-cadherin and endogenous β-catenin (Figure 9G). In contrast, the R317C, K623*, or R700Q variants showed remarkably compromised interactions with both VE-cadherin and β-catenin (Figure 9G), indicating that these variants in *CTNND1* caused a disrupted cadherin/catenin complex, which could result in degradation of β-catenin and compromised signaling transduction.

### p120 genetically interacts with β-catenin and α-catenin.

We have previously reported that EC-specific knockout of *Ctnna1* (α-catenin) in mice causes FEVR-like phenotypes through disruption of the cadherin/catenin complex and abnormal overactivation of β-catenin signaling (20). However, in this paper, we observed disruption of the cadherin/catenin complex and inactivation of β-catenin signaling in EC-specific *Ctnnd1*-knockout mice and *CTNND1*-depleted HRECs. We therefore asked whether *Ctnnd1* knockout in murine ECs could result in distinct phenotypes from those of *Ctnna1*-knockout mice. Aligned comparisons of these retinas showed that, despite both models exhibiting vascular hyperplasia and extensive leakage of erythrocytes in the peripheral areas of the superficial retinal vasculature, *Ctnnd1^iECKO/iECKO^* mice exhibited sparsely distributed vasculature in the remodeling plexus compared with the extreme hyperplasia seen in the *Ctnna1^iECKO/iECKO^* case (Supplemental Figure 9). This discrepancy might be partially due to the opposite roles that α-catenin and p120 play in the regulation of Wnt/β-catenin signaling. We constructed *Ctnnb1^iECKO/iECKO^* mice, which exhibited retinal vascular phenotypes similar to those of *Ctnnd1^iECKO/iECKO^* mice, except with less leakage. This indicated that disruption of AJs might play a pivotal role in the maintenance of vascular integrity (Supplemental Figure 9).

**Figure 10:**
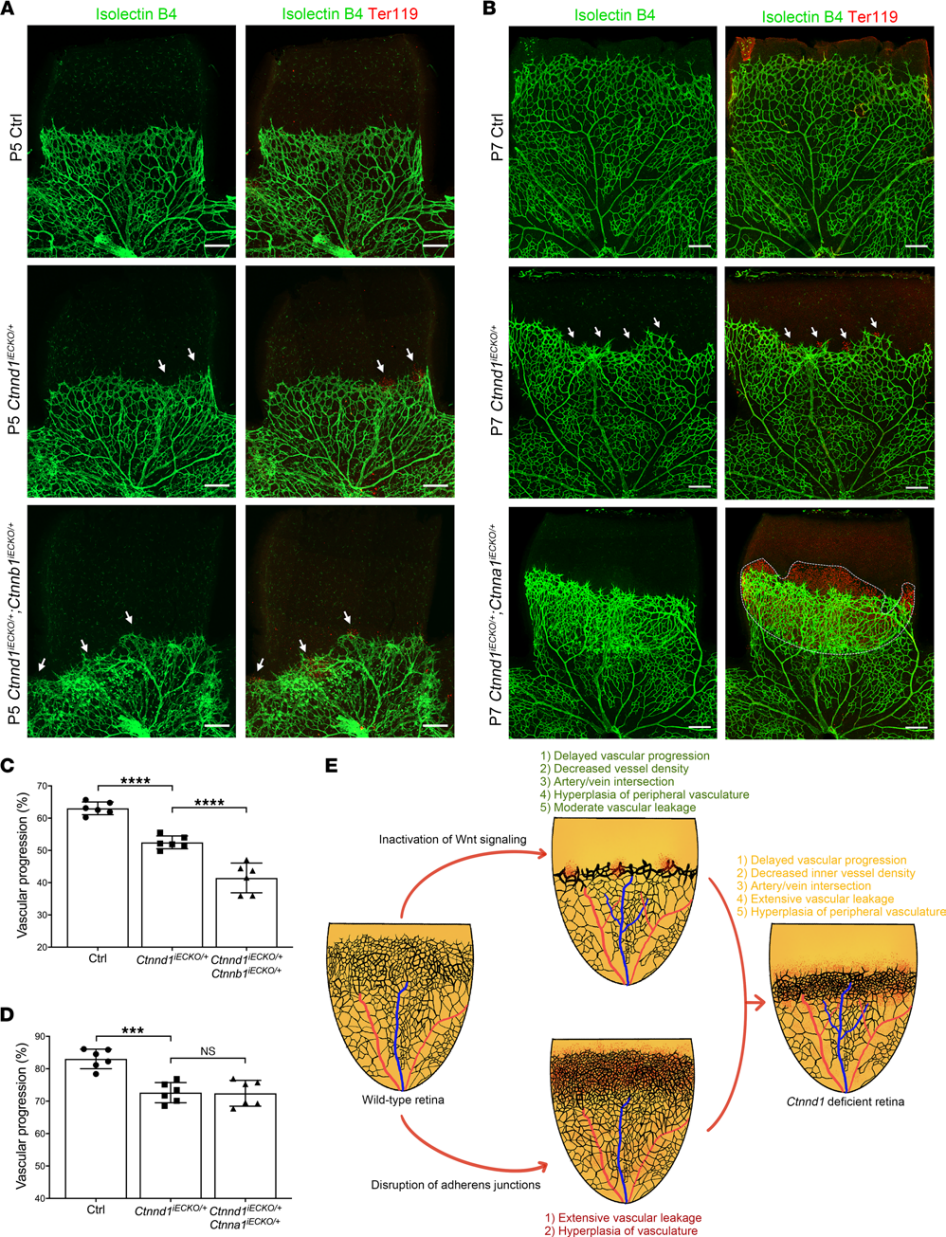
FEVR-like phenotypes in double-heterozygous deletion of *Ctnnd1* and *Ctnna1* or *Ctnnb1* in mouse ECs and schematic diagram of the mechanism for p120 deficiency in the pathogenesis of FEVR. (**A**) Representative immunofluorescence images of P5 Ctrl, *Ctnnd1^iECKO/+^*, and *Ctnnd1^iECKO/+^* *Ctnnb1^iECKO/+^* mouse retinas labeled with IB4 (green) and Ter119 (red). The white arrows indicate abnormal leakage of erythrocytes. Scale bars, 200 μm. (**B**) Representative immunofluorescence images of P7 Ctrl, *Ctnnd1^iECKO/+^*, and *Ctnnd1^iECKO/+^* *Ctnna1^iECKO/+^* mouse retinas labeled with IB4 (green) and Ter119 (red). The white arrows and dotted region indicate abnormal leakage of erythrocytes. Scale bars, 200 μm. (**C**) Quantification of vascular progression of P5 Ctrl, *Ctnnd1^iECKO/+^*, and *Ctnnd1^iECKO/+^* *Ctnnb1^iECKO/+^* mouse retinas. Error bars, SDs. The *P* values are from multiple comparisons in 1-way ANOVA with Dunnett’s multiple comparisons tests (*n* = 6); ****P < 0.0001. (**D**) Quantification of vascular progression of P7 Ctrl, *Ctnnd1^iECKO/+^*, and *Ctnnd1^iECKO/+^* *Ctnna1^iECKO/+^* mouse retinas. Error bars, SDs. The *P* values are from multiple comparisons in 1-way ANOVA with Dunnett’s multiple comparisons tests (*n* = 6); ***P < 0.001. (**E**) Inactivation of Wnt signaling (upper panel) and disruption of AJs (lower panel) lead to similar but distinct retinal vascular defects. Deficient p120 function causes FEVR through combined effects of Wnt inactivation and AJ disruption (right panel). Inactivation of Wnt signaling contributes to i) delayed vascular progression, ii) decreased inner vessel density, iii) artery/vein intersection, iv) hyperplasia of peripheral vasculature, and v) moderate vascular leakage (upper panel). Disruption of AJs contributes to i) extensive vascular leakage and ii) vascular hyperplasia (lower panel). Experiments were performed at least 3 times independently.

We further bred double-heterozygous knockout mice that each lacked 1 allele of *Ctnnd1*, *Ctnna1*, or *Ctnnb1* to elucidate whether p120 genetically interacts with α-catenin or β-catenin. We crossed *Ctnnd1^iECKO/+^* mice with either *Ctnna1^fl/+^* or *Ctnnb1^fl/+^* mice to generate wild-type, single-gene heterozygous, or double-heterozygous offspring. Notably, the vasculature of *Ctnnd1^iECKO/+^* mouse retinas showed moderate FEVR-like phenotypes, including delayed superficial vascular progression, leakage of erythrocytes, and crossings of arteries and veins (Figure 10). Further deleting 1 allele of the *Ctnnb1* in *Ctnnd1^iECKO/+^* mouse ECs led to increased vessel leakage, artery/vein crossings, and peripheral vascular hyperplasia, as well as more severely compromised vascular progression compared with *Ctnnd1^iECKO/+^* mice (Figure 10, A and C), which closely recapitulated the phenotypes observed in *Ctnnd1^iECKO/iECKO^* mice. Deletion of 1 *Ctnna1* allele from *Ctnnd1^iECKO/+^* mice caused severe vascular leakage and hyperplasia in the leakage areas, whereas the vascular progression was undisturbed (Figure 10, B and D).

We next tested immune responses, including astrogliosis and microgliosis, in these double-heterozygous knockout mice. As expected, both *Ctnnd1^iECKO/+^* *Ctnnb1^iECKO/+^* and *Ctnnd1^iECKO/+^* *Ctnna1^iECKO/+^* mouse retinas exhibited increased GFAP intensity in the angiogenic front and remodeling plexus compared with that of controls (Supplemental Figure 10), indicative of reactive astrogliosis in the double-heterozygous knockout mouse retinas. In the angiogenic front, *Ctnnd1^iECKO/+^* *Ctnna1^iECKO/+^* mouse retinas showed higher GFAP levels compared with those of *Ctnnd1^iECKO/+^* *Ctnnb1^iECKO/+^* mice (Supplemental Figure 10, A and B), whereas the GFAP levels were comparable in the remodeling plexus of *Ctnnd1^iECKO/+^* *Ctnna1^iECKO/+^* and *Ctnnd1^iECKO/+^* *Ctnnb1^iECKO/+^* mouse retinas (Supplemental Figure 10, A and C). Moreover, although the number of IBA1-expressing microglia was comparable in *Ctnnd1^iECKO/+^* *Ctnna1^iECKO/+^* and *Ctnnd1^iECKO/+^* *Ctnnb1^iECKO/+^* mouse retinas, they were remarkably increased with amoeboid shape transformation compared with those of control mice (Supplemental Figure 11).

## Discussion

p120 is an indispensable component of the cadherin/catenin complex that participates in the normal function of several tissues and organ systems (54–56). However, the regulatory role of p120 in postnatal retinal vascular development remains unclear due to the embryonic lethality of *Tie2-Cre*–mediated knockout of *Ctnnd1* in the early embryonic stage (33). In this study, using WES on the genomic DNA of probands in 140 FEVR-associated families without known genetic variants, we identified 3 heterozygous variants in the *CTNND1* gene, including 1 nonsense and 2 missense variants.

Interestingly, the proband and his affected mother in Family-506, who were heterozygous for the K623* variant, manifested distinct features of BCD syndrome phenotypes in addition to FEVR phenotypes (29–32). BCD syndrome is a rare disorder characterized by typical manifestations of CLP, eyelid malformations, and ectodermal defects, as well as other atypical manifestations of hypothyroidism, imperforate anus, neural tube defects, and syndactyly. However, the FEVR-associated phenotype has not yet been reported in patients with BCD syndrome to our knowledge (29–32). *CTNND1* variants have been associated with BCD syndrome. Notably, of this and the 15 previously reported variants in the *CTNND1* gene that cause BCD syndrome, 13 were frameshift or nonsense variants, indicating that p120 participates in different aspects of human development. Frameshift variants with severe disruption of p120 function might cause systemic development defects, which is consistent with the observation that the K623* variant causes BCD syndrome phenotypes in addition to FEVR (29–32).

In vitro functional studies indicated that all variants compromised the Wnt signaling activity through disruption of interactions between p120 and VE-cadherin or β-catenin (Figure 9). Thus, we conclude that these variants are candidate pathogenic variants capable of causing FEVR through disruption of the cadherin/catenin complex and inactivation of Wnt/β-catenin signaling. Intriguingly, depletion of *Ctnnd1* in the postnatal mouse ECs largely phenocopied typical manifestations of FEVR, confirming that *CTNND1* could be a candidate gene for FEVR.

To investigate the mechanism by which p120 regulates EC function, we applied an unbiased proteomics analysis of *CTNND1*-knockdown HRECs and found prominent enrichment of differentially expressed proteins in the cadherin and Wnt signaling pathways (Figure 5, A and B). It has been previously reported that knockdown of *CTNND1* in human dermal microvascular ECs results in downregulation of cadherin/catenin components (22). Additionally, overexpression of *CTNND1* in HUVECs significantly increases the levels of VE-cadherin (57). As expected, in the *CTNND1*-knockdown HRECs, we found disruption of the AJs and downregulation of cadherin/catenin components, including α-catenin, β-catenin, and VE-cadherin, which were regulated at the protein level, rather than at the mRNA level (Figures 5 and 6).

The role of p120 in Wnt signaling in various cells or organs remains controversial. In prostate cancer cell lines, p120 has been reported to activate the Wnt/β-catenin signaling pathway (58). However, p120 has a negative role in regulating the Wnt/β-catenin signaling pathway in epithelia (59). Additionally, it has been reported to be redundant in regulating the Wnt/β-catenin signaling pathway in human corneal ECs (60). Here, we showed that p120 functioned as an activator of the Wnt/β-catenin signaling pathway; loss of p120 resulted in degradation of β-catenin and further downregulated genes involved downstream of the Wnt/β-catenin signaling pathway.

In mouse retinas, vasculature formed from vessel sprouting subsequently undergoes vessel pruning from P5~P18 to form a functionally mature network (61). The Wnt/β-catenin pathway has been widely studied in the regulation of vessel pruning. Loss of endothelial *Ctnnb1* or *Lef1* in mice has been reported to cause premature vessel regression (61, 62). In contrast, transgenic gain of *Ctnnb1* function in mouse ECs or global deletion of a negative Wnt/β-catenin regulator *Apcdd1* resulted in activation of Wnt/β-catenin signaling and delayed vessel pruning (63, 64). Likewise, loss of Semaphorin 3G or the endothelial transcription factor ERG led to decreased Wnt/β-catenin signaling and increased vessel pruning in the mouse retinas (41, 46). Thus, it is reasonable to speculate that the abnormally increased vessel pruning in *Ctnnd1^iECKO/iECKO^* mouse retinas was a consequence of compromised β-catenin signaling (Supplemental Figure 4D). Furthermore, it is worth noting that although the lack of p120 in ECs led to compromised Wnt signaling and increased vessel pruning, the cell proliferation and vascular density in the angiogenic fronts of *Ctnnd1^iECKO/iECKO^* mouse retinas were abnormally elevated. We hypothesized and supported that, in the angiogenic front, deletion of endothelial *Ctnnd1* leads to extensive vessel leakage, which stimulates immune responses and excessive Vegfa secretion, as well as increased sensitivity of ECs to Vegfa, which bypasses the negative effect of compromised Wnt signaling and collectively contributes to hyperplasia in the angiogenic front.

Interestingly, treatment with LiCl or CHIR-99021, which are inhibitors of Gsk3β, considerably restored Wnt/β-catenin signaling activity and promoted the in vitro proliferation of p120-depleted HRECs (Supplemental Figure 7). This indicates that p120 might protect β-catenin from Gsk3β-mediated ubiquitination degradation. It is notable that although LiCl treatment of *Ctnnd1^iECKO/iECKO^* mice considerably increased the vascular density of their retinas (possibly due to enhanced β-catenin signaling and inhibited pruning), LiCl failed to rescue compromised vascular progression and BRB integrity, suggesting that disrupted cadherin/catenin complexes play nonnegligible roles in the progress of FEVR-like phenotypes in *Ctnnd1^iECKO/iECKO^* mice. These results further highlighted the role of normal AJ integrity in retinal vascularization, which might be a therapeutic target for FEVR.

Genetic and functional analyses revealed that heterozygous variants in the *CTNND1* gene caused FEVR in a presumed autosomal dominant manner of inheritance, consistent with the fact that most *FZD4*, *LRP5*, and *TSPAN12* variants are inherited as autosomal dominant FEVR (10, 34, 65). However, mice subjected to heterozygous global knockout of *Fzd4*, *Lrp5*, or *Tspan12* exhibit normal vascularization (10, 34, 65). In contrast, heterozygous knockout of *Ctnnd1* in mouse ECs manifested moderate FEVR-like phenotypes (Figure 10), indicating that angiogenesis is more sensitive to the *Ctnnd1* dosage than to the dosages of the abovementioned Wnt-related genes (10, 34, 65). We hypothesized that the disruption of the cadherin/catenin complex, in addition to compromised Wnt/β-catenin signaling, might be the mechanism of the haploinsufficiency of *CTNND1* in the pathogenesis of FEVR.

Using structural and functional analyses, we revealed that variants in *CTNND1* compromised the p120/VE-cadherin and p120/β-catenin interactions (Figure 9, F and G). It is worth noting that p120 has been reported to indirectly interact with β-catenin or α-catenin (66); thus, we speculate that the compromised interactions between p120 variants and β-catenin were largely due to the compromised p120/cadherin interaction. Moreover, although it is sufficient to conclude that the K623* and R700Q variants compromised p120/VE-cadherin interaction by disrupting the binding surfaces in the ARM domains of p120, further investigation of the mechanism by which the R317C variant affects interactions between p120 and VE-cadherin is limited by the lack of a 3-dimensional structure of the full-length p120/VE-cadherin complex.

In our previous research, we showed that conditional knockout of *Ctnna1* in ECs resulted in FEVR-like vascular defects and a radically distinct vascular density compared with that of *Fzd4*- or *Lrp5*-knockout mice (20). Here, *Ctnna1^iECKO/iECKO^* mice exhibited extensive hyperplasia compared with the sparsely distributed vasculature in the remodeling plexus of *Ctnnd1^iECKO/iECKO^* mouse retinas (Supplemental Figure 9) (20). These observations indicate that, although p120 and α-catenin are indispensable components of the cadherin/catenin complex associated with FEVR, they may act through distinct mechanisms.

It has been reported that α-catenin inhibits Wnt signaling by segregating the available pool of β-catenin in cytosol, and that variants of α-catenin disrupt the cadherin/catenin complex that releases β-catenin from cytosol for nuclear translocation and activation of β-catenin signaling, which may contribute to the pathogenesis of FEVR (20, 67–70). Notably, we observed genetic interactions between p120 and β-catenin or α-catenin in mice (Figure 10), albeit with distinguishing manifestations. This discrepancy could be explained by the hypothesis that double-heterozygous loss of *Ctnnd1* and *Ctnna1* prominently contributes to compromised AJ integrity, leading to severe vascular leakage and associated immune responses that induce hyperplasia without affecting vascular progression, whereas double-heterozygous deletion of *Ctnnd1* and *Ctnnb1* impairs vascular development prominently through inactivation of Wnt signaling, thus leading to more typical FEVR-like phenotypes.

Taken together, we demonstrate that variants in the *CTNND1* gene cause FEVR through the combined contributions of AJ disruption and Wnt signaling inactivation (Figure 10E). This offers potentially novel pathogenic clues concerning the development of FEVR and other diseases involving the vascular system.

## Methods

A full description of the methods is presented in the online-only Supplemental Methods.

### Mouse models.

*Ctnnd1* conditional knockout mice (*Ctnnd1^tm1Lfr^*/Mmucd) (71) were purchased from the Mutant Mouse Resource & Research Centers and bred into a background of *Pdgfb-iCre-ER* mice (72). The *Ctnna1^iECKO/iECKO^* model was constructed as previously described (20). The *Ctnnb1^loxP/loxP^* mice were obtained from The Jackson Laboratory (stock number 004152).

### Clinical data.

Patients and their family members were enrolled and evaluated by ophthalmologists at Zhongshan Ophthalmic Center, Sun Yat-sen University, and Xinhua Hospital, Shanghai Jiaotong University.

### Plasmids.

Wild-type *CTNND1* coding sequence was subcloned into a mammalian expression vector with N-terminal FLAG-tagged pCDNA3.1 vector (both from Youbio Biotechnology). *CTNND1* variants were generated by site-directed mutagenesis using a Q5 Site-Directed Mutagenesis Kit (E0554S, New England Biolabs) and confirmed by Sanger sequencing.

### Image acquisition.

The microscope (LSM 900, Zeiss) was used to capture confocal images. The Spline Contour2 and line tools in Zen 2.1 software (Zeiss) were used to evaluate intensity mean value of cell junction proteins and vascular progression of whole mounts, respectively. Angiotool software was used to measure the percentage of vessel area as previously described (73). The immunoblot signals were detected by ChemiDoc (Bio-Rad) and analyzed by ImageJ software (NIH).

### Data and materials availability.

All the data and materials are available within the article and the supplemental materials. Supplemental data include 11 figures and 6 tables.

### Statistics.

Statistical analysis was performed with GraphPad Prism 8.0. To assess statistical significance with a 95% confidence level, unpaired 2-tailed Student’s *t* test with Welch’s correction was applied to comparisons between 2 experimental groups, while 1-way ANOVA or 2-way ANOVA with Dunnett’s or Tukey’s multiple comparison tests were performed in multiple comparisons among more than 2 experimental groups. All the experiments were conducted at least 3 times independently. *P* < 0.05 was considered statistically significant.

### Study approval.

The clinical study was conducted in accordance with the declaration of Helsinki and approved by the ethical oversight committee of Sichuan Provincial People’s Hospital, Zhongshan Ophthalmic Center, Sun Yat-sen University, and Xinhua Hospital, Shanghai Jiaotong University. Written informed consent was obtained from all participants or legal guardians for minors. The Institutional Animal Care and Use Committee at the Sichuan Provincial People’s Hospital approved all animal study protocols. All experimental procedures were carried out according to the approved protocols.

## Author contributions

XZ, ZY, and XD conceived the research. MY, SL, and RZ performed the animal model study and cell biology, immunohistochemistry, and gene expression studies. LH, XJ, YH, JL, LP, ED, WL, ZZ, DJ, YZ, ZJ, and YY performed the construction of plasmids and animal breeding. XD, LH, and PZ recruited the participants and performed the WES study and Sanger sequencing analysis. MY, SL, XD, and XZ analyzed data. MY, SL, and XZ wrote the manuscript, with input from other authors.

## Supplementary Material

Supplemental data

Supplemental table 1

## Version 1 06/14/2022

In-Press Preview

## Version 2 07/22/2022

Electronic publication

## References

[1] Su W, Kowalczyk AP (2017). The VE-cadherin cytoplasmic domain undergoes proteolytic processing during endocytosis. Mol Biol Cell.

[2] Mayor R, Etienne-Manneville S (2016). The front and rear of collective cell migration. Nat Rev Mol Cell Biol.

[3] Carmeliet P, Jain RK (2011). Principles and mechanisms of vessel normalization for cancer and other angiogenic diseases. Nat Rev Drug Discov.

[4] Fallah A (2019). Therapeutic targeting of angiogenesis molecular pathways in angiogenesis-dependent diseases. Biomed Pharmacother.

[5] Criswick VG, Schepens CL (1969). Familial exudative vitreoretinopathy. Am J Ophthalmol.

[6] Fei P (2021). Early detection of ocular abnormalities in a Chinese multicentre neonatal eye screening programme-1-year result. Acta Ophthalmol.

[7] Robitaille J (2002). Mutant frizzled-4 disrupts retinal angiogenesis in familial exudative vitreoretinopathy. Nat Genet.

[8] Toomes C (2004). Mutations in LRP5 or FZD4 underlie the common familial exudative vitreoretinopathy locus on chromosome 11q. Am J Hum Genet.

[9] Jiao X (2004). Autosomal recessive familial exudative vitreoretinopathy is associated with mutations in LRP5. Am J Hum Genet.

[10] Junge HJ (2009). TSPAN12 regulates retinal vascular development by promoting Norrin- but not Wnt-induced FZD4/beta-catenin signaling. Cell.

[11] Nikopoulos K (2010). Next-generation sequencing of a 40 Mb linkage interval reveals TSPAN12 mutations in patients with familial exudative vitreoretinopathy. Am J Hum Genet.

[12] Poulter JA (2010). Mutations in TSPAN12 cause autosomal-dominant familial exudative vitreoretinopathy. Am J Hum Genet.

[13] Chen ZY (1993). A mutation in the Norrie disease gene (NDP) associated with X-linked familial exudative vitreoretinopathy. Nat Genet.

[14] Li S Variants in the Wnt co-receptor LRP6 are associated with familial exudative vitreoretinopathy. J Genet Genomics.

[15] Dixon MW (2016). CTNNB1 mutation associated with familial exudative vitreoretinopathy (FEVR) phenotype. Ophthalmic Genet.

[16] Panagiotou ES (2017). Defects in the cell signaling mediator β-catenin cause the retinal vascular condition FEVR. Am J Hum Genet.

[17] He Y Novel truncating variants in CTNNB1 cause familial exudative vitreoretinopathy. J Med Genet.

[18] Park H (2019). Integrin-linked kinase controls retinal angiogenesis and is linked to Wnt signaling and exudative vitreoretinopathy. Nat Commun.

[19] Zhang S (2021). Whole-exome sequencing identified DLG1 as a candidate gene for familial exudative vitreoretinopathy. Genet Test Mol Biomarkers.

[20] Zhu X (2021). Catenin α 1 mutations cause familial exudative vitreoretinopathy by overactivating Norrin/β-catenin signaling. J Clin Invest.

[21] Zhao JL (2019). Knockdown of P120 catenin aggravates endothelial injury under an impinging flow by inducing breakdown of adherens junctions. Mol Med Rep.

[22] Herron CR (2011). p120 regulates endothelial permeability independently of its NH2 terminus and Rho binding. Am J Physiol Heart Circ Physiol.

[23] Nanes BA (2017). p120-catenin regulates VE-cadherin endocytosis and degradation induced by the Kaposi sarcoma-associated ubiquitin ligase K5. Mol Biol Cell.

[24] Ishiyama N (2018). Force-dependent allostery of the α-catenin actin-binding domain controls adherens junction dynamics and functions. Nat Commun.

[25] Nanes BA (2012). p120-catenin binding masks an endocytic signal conserved in classical cadherins. J Cell Biol.

[26] Grimsley-Myers CM (2020). VE-cadherin endocytosis controls vascular integrity and patterning during development. J Cell Biol.

[27] Kourtidis A (2013). p120 catenin: an essential regulator of cadherin stability, adhesion-induced signaling, and cancer progression. Prog Mol Biol Transl Sci.

[28] Casagolda D (2010). A p120-catenin-CK1epsilon complex regulates Wnt signaling. J Cell Sci.

[29] Cox LL (2018). Mutations in the epithelial cadherin-p120-catenin complex cause mendelian non-syndromic cleft lip with or without cleft palate. Am J Hum Genet.

[30] Alharatani R (2020). Novel truncating mutations in CTNND1 cause a dominant craniofacial and cardiac syndrome. Hum Mol Genet.

[31] Kievit A (2018). Variants in members of the cadherin-catenin complex, CDH1 and CTNND1, cause blepharocheilodontic syndrome. Eur J Hum Genet.

[32] Ghoumid J (2017). Blepharocheilodontic syndrome is a CDH1 pathway-related disorder due to mutations in CDH1 and CTNND1. Genet Med.

[33] Oas RG (2010). p120-catenin is required for mouse vascular development. Circ Res.

[34] Xu Q (2004). Vascular development in the retina and inner ear: control by Norrin and Frizzled-4, a high-affinity ligand-receptor pair. Cell.

[35] Fruttiger M (2007). Development of the retinal vasculature. Angiogenesis.

[36] Wang Y (2012). Norrin/Frizzled4 signaling in retinal vascular development and blood brain barrier plasticity. Cell.

[37] Luhmann UF (2005). Role of the Norrie disease pseudoglioma gene in sprouting angiogenesis during development of the retinal vasculature. Invest Ophthalmol Vis Sci.

[38] Wang Z (2019). Wnt signaling in vascular eye diseases. Prog Retin Eye Res.

[39] Seymour PA (2020). Jag1 modulates an oscillatory Dll1-notch-Hes1 signaling module to coordinate growth and fate of pancreatic progenitors. Dev Cell.

[40] Wang Y (2020). A mouse model for kinesin family member 11 (Kif11)-associated familial exudative vitreoretinopathy. Hum Mol Genet.

[41] Chen DY (2021). Endothelium-derived semaphorin 3G attenuates ischemic retinopathy by coordinating β-catenin-dependent vascular remodeling. J Clin Invest.

[42] Xiao K (2005). p120-Catenin regulates clathrin-dependent endocytosis of VE-cadherin. Mol Biol Cell.

[43] Veys K (2020). Role of the GLUT1 glucose transporter in postnatal CNS angiogenesis and blood-brain barrier integrity. Circ Res.

[44] Zhou T (2017). Microglia polarization with M1/M2 phenotype changes in rd1 mouse model of retinal degeneration. Front Neuroanat.

[45] Gerhardt H (2003). VEGF guides angiogenic sprouting utilizing endothelial tip cell filopodia. J Cell Biol.

[46] Birdsey GM (2015). The endothelial transcription factor ERG promotes vascular stability and growth through Wnt/β-catenin signaling. Dev Cell.

[47] Ye X (2009). Norrin, frizzled-4, and Lrp5 signaling in endothelial cells controls a genetic program for retinal vascularization. Cell.

[48] Yang M (2021). The ER membrane protein complex subunit Emc3 controls angiogenesis via the FZD4/WNT signaling axis. Sci China Life Sci.

[49] Nusse R, Clevers H (2017). Wnt/β-catenin signaling, disease, and emerging therapeutic modalities. Cell.

[50] Valenta T (2012). The many faces and functions of β-catenin. EMBO J.

[51] Stan RV (1999). PV-1 is a component of the fenestral and stomatal diaphragms in fenestrated endothelia. Proc Natl Acad Sci U S A.

[52] Ishiyama N (2010). Dynamic and static interactions between p120 catenin and E-cadherin regulate the stability of cell-cell adhesion. Cell.

[53] Ireton RC (2002). A novel role for p120 catenin in E-cadherin function. J Cell Biol.

[54] Davis MA, Reynolds AB (2006). Blocked acinar development, E-cadherin reduction, and intraepithelial neoplasia upon ablation of p120-catenin in the mouse salivary gland. Dev Cell.

[55] Kurley SJ (2012). p120-catenin is essential for terminal end bud function and mammary morphogenesis. Development.

[56] Marciano DK (2011). p120 catenin is required for normal renal tubulogenesis and glomerulogenesis. Development.

[57] Alcaide P (2008). p120-catenin regulates leukocyte transmigration through an effect on VE-cadherin phosphorylation. Blood.

[58] He Y (2015). δ-catenin interacts with LEF-1 and negatively regulates its transcriptional activity. Cell Biol Int.

[59] Hernandez-Martinez R (2019). p120-catenin regulates WNT signaling and EMT in the mouse embryo. Proc Natl Acad Sci U S A.

[60] Zhu YT (2012). Nuclear p120 catenin unlocks mitotic block of contact-inhibited human corneal endothelial monolayers without disrupting adherent junctions. J Cell Sci.

[61] Korn C, Augustin HG (2015). Mechanisms of vessel pruning and regression. Dev Cell.

[62] Phng LK (2009). Nrarp coordinates endothelial Notch and Wnt signaling to control vessel density in angiogenesis. Dev Cell.

[63] Corada M (2010). The Wnt/beta-catenin pathway modulates vascular remodeling and specification by upregulating Dll4/Notch signaling. Dev Cell.

[64] Mazzoni J (2017). The Wnt inhibitor Apcdd1 coordinates vascular remodeling and barrier maturation of retinal blood vessels. Neuron.

[65] Lobov IB (2005). WNT7b mediates macrophage-induced programmed cell death in patterning of the vasculature. Nature.

[66] Daniel JM, Reynolds AB (1995). The tyrosine kinase substrate p120cas binds directly to E-cadherin but not to the adenomatous polyposis coli protein or alpha-catenin. Mol Cell Biol.

[67] Carmeliet P (1999). Targeted deficiency or cytosolic truncation of the VE-cadherin gene in mice impairs VEGF-mediated endothelial survival and angiogenesis. Cell.

[68] Hwang SG (2005). Regulation of beta-catenin signaling and maintenance of chondrocyte differentiation by ubiquitin-independent proteasomal degradation of alpha-catenin. J Biol Chem.

[69] Simcha I (1998). Differential nuclear translocation and transactivation potential of beta-catenin and plakoglobin. J Cell Biol.

[70] Takahashi N (2000). Posttranscriptional regulation of alpha-catenin expression is required for Wnt signaling in L cells. Biochem Biophys Res Commun.

[71] Elia LP (2006). p120 catenin regulates dendritic spine and synapse development through Rho-family GTPases and cadherins. Neuron.

[72] Pitulescu ME (2010). Inducible gene targeting in the neonatal vasculature and analysis of retinal angiogenesis in mice. Nat Protoc.

[73] Zudaire E (2011). A computational tool for quantitative analysis of vascular networks. PLoS One.

