# Supplementary material for: CTNND1 variants cause familial exudative vitreoretinopathy through the Wnt/cadherin axis
Source: JCI Insight. 2022 Jul 22;7(14):e158428. doi: 10.1172/jci.insight.158428 (PMC9431724; doi:10.1172/jci.insight.158428)
Supplement: Supplemental data [file jciinsight-7-158428-s043.pdf]

# Supplementary Materials for

# *CTNND1* variants cause familial exudative vitreoretinopathy through Wnt/Cadherin axis

Mu Yang[1,2#], Shujin Li[1,2#], Li Huang[3#], Rulian Zhao[1#], Erkuan Dai[4], Xiaoyan Jiang[1], Yunqi He[1], Jinglin Lu[3], Li Peng[1], Wenjing Liu[1], Zhaotian Zhang[3], Dan Jiang[1], Yi Zhang[1], Zhilin Jiang[1], Yeming Yang[1], Peiquan Zhao[4], Xianjun Zhu[1,2,5*], Xiaoyan Ding[3*], Zhenglin Yang[1,2*]

Supplemental data include Supplemental Material and Methods, supplemental figures S1-S11, tables S2-S6.

## Supplemental Material and Methods

### Whole-Exome Sequencing

Genomic DNA of the proband and available family members was extracted from peripheral blood leukocytes obtained from 140 FEVR families and 1000 control individuals using the TIANamp Blood DNA Kit (Tiangen Biotech, Beijing, China). The quantity and quality of DNA were verified by using NanoDrop. Illumina paired-end libraries were prepared using Kapa LTP library prep kit (Roche, Basel, Switzerland) according to the protocol provided by the manufacturer. Briefly, genomic DNA was sheared into fragments of approximately 300-500 bp in length. The DNA fragments were end-repaired, and an extra 'adenine' base was added to the 3' end. Illumina adapters were ligated to the ends of the DNA fragments and four cycles of PCR amplification were applied to each sample after ligation. The DNA libraries were quantified by the Qubit 3.0. Precapture libraries were pooled together for one capture reaction. Agilent SSELXT Human All Exon V6 was used for whole-exome sequencing (Agilent, Santa Clara, CA, USA). The enriched DNA library was sequenced on Illumina X Ten Analyzers for 150 cycles per read to generate paired-end reads following the manufacturer's standard sequencing protocols.

The raw sequence data reported in this paper have been deposited in the Genome Sequence Archive in National Genomics Data Center (1), Beijing Institute of Genomics (China National Center for Bioinformation), Chinese Academy of Sciences, under accession number HRA001957 that are publicly accessible at https://bigd.big.ac.cn/gsa.

### Bioinformatics Analysis of Sequencing Results

The high-quality sequencing reads were aligned to the human genome reference (NCBI build 37.1/hg19) using the BWA (Burrows Wheeler Aligner). Single-nucleotide variants (SNVs) and InDels (Insertions and Deletions) were analyzed by GATK4.0 HC. SNVs and InDels were detected only on exome regions and on flanking regions within size of 200 bp. The detected genomic variants were mapped and filtered using the

dbSNP151, Exome Variant Server, ExAC, 1000 Genome, and gnomAD databases and the internal database of Clinbytes Inc, with an allele frequency cutoff value as 0.5% and 0.1% for recessive and dominant variants, respectively.

The detected variants in *CTNND1* were validated in all available family members by Sanger sequencing. Primers for variant validation by Sanger sequencing were listed in supplementary Table S2. PCR products were sequenced on an ABI 3700XL Genetic Analyzer.

**Mouse models and genotyping**

The mice were maintained under the 12-hour light/dark cycle with ad-lib diet. To induce Cre activity, newborn littermates were intraperitoneally injected daily with 50 μg Tamoxifen (consecutive 3 days from postnatal day 1-3). In all animal experiments, Tamoxifen-injected $Ctnnd1^{loxP/loxP}$ or *Pdgfb-iCre-ER* mice were used as control. Primers for genotyping were shown in supplementary Table S3.

**Electron microscopy**

The eyes were prefixed with fixative solution (4% paraformaldehyde and 2.5% glutaraldehyde), then fixed in 1% osmium tetroxide, dehydrated with acetone, and embedded with Epon 812. The procedures of semithin sections, ultrathin sections, and examination were applied as previously described (2).

**Immunofluorescence staining, hematoxylin-eosin staining, EdU labeling and paracellular integrity analysis of mice retinas**

The intact eyeballs were fixed in 4% PFA for 20 min at room temperature followed by 20 min rinse with PBS. Dissection of whole-mounted retinas and isolation of hyaloid vessels were carried out as described previously (3). The dissected retinas were fixed in methanol at -20 °C before rinse with PBS. For retinal frozen sections, enucleated eyes were fixed in 4% PFA for 2h at 4 °C following with dehydration and embedded in optimal cutting temperature compound (Sakura Finetek). For hematoxylin and eosin staining of paraffin sections, the fixed eyes were transferred into 70% ethanol and embedded in paraffin. Paraffin sections were obtained using a paraffin microtome, and

deparaffinized with xylene, dehydrated through graded series of ethanol, followed by staining with hematoxylin and eosin. Human retinal microvascular endothelial cells (HRECs, Cell Systems, catalog # ACBRI-181) were cultured in EGM-2 BulletKit medium (Lonza, catalog # CC-3202) and seeded on 5 μg/ml human fibronectin (Thermo Fisher Scientific, catalog # 33016015) coated slices. Cells before passage seven were used in all the experiments. After being fixed in 4% PFA for 15 min at room temperature, the slides were rinsed with PBS for 3 times and blocked with 5% fetal bovine serum (Gibco, catalog # 10100147C) for 30 min at room temperature. For immunofluorescence staining, the whole-mount, retinal sections or cells were incubated with primary antibodies overnight at 4 ℃ and underwent three washes with PBS followed with incubation in secondary antibodies diluted in PBS at room temperature. The antibodies used for retinal immunofluorescence staining were shown in Table S4.

For labeling of developing vascular endothelial cells in the retinas, 50 μl of 2 mM EdU was intraperitoneally injected at P6 for 3h before mice sacrifice. To detect the proliferation of HRECs, 10 μM EdU were pretreated for 3h. The EdU-positive cells were then stained using Click-iT EdU Alexa Fluor-594 Imaging Kit (C10339; Invitrogen).

For paracellular junctional integrity analysis, 2% fluorescent tracer, 5-(and-6)-tetramethylrhodamine biocytin, biocytin-TMR (MW = 869 Da, CAT#T12921 Thermo Fisher Scientific) was intraperitoneally injected 24h prior to sacrifice (4).

**Lentivirus mediated knockdown and Matrigel assay**

Lentivirus carrying s*h-RNA* targeting *CTNND1* *(5'*-CAGCCAGAGGTGGTTCGGATATACA-*3')* and negative-control *sh-RNA* (*5'*-TTCTCCGAACGTGTCACGT-*3'*) were used for knockdown of *CTNND1.* Lentivirus for knockdown of *CTNNA1* was constructed as previously described (2). A μ-slide (81506, Ibidi) was coated with 10 μl ice-cold Matrigel (354234, BD Pharmingen) and subsequently incubated at 37 ℃ for half an hour. HRECs were trypsinized and plated on the Matrigel at $1\times10^5$ cells/well, followed by 37 ℃ 5% $CO_2$ incubation for 3 h.

**Mass spectrometry and proteomics analysis**

HRECs in the lysis buffer (8 M urea, 1% Protease Inhibitor Cocktail) were sonicated three times on ice and the debris was removed by centrifugation at 12,000 g at 4 °C for 10 min. The remaining supernatant was measured using Bradford reagent (Sigma-Aldrich, St. Louis, MO, USA). The protein solution, which was reduced with 5 mM dithiothreitol and alkylated with 11 mM iodoacetamide, was diluted to urea concentration less than 2 M with100 mM TEAB. Finally, trypsin was added for the first digestion overnight (1:50 trypsin-to-protein mass ratio) and the second 4-hour-digestion (1:100 trypsin-to-protein mass ratio). The tryptic peptides were dissolved in 0.1% formic acid (solvent A) and label-free quantitation (LFQ) mass spectrometry was performed on Q ExactiveTM Plus (Thermo) coupled online to the UPLC (5). The resolution of 70,000 was utilized for MS and 20 MS/MS spectra were acquired per MS scan. The data were identified and analyzed through bioinformatics software package as described (6). Gene pathway classification was carried out using PANTHER (www.pantherdb.org) (7). The input gene was selected following the three criteria: 1) The significance should be $p<0.05$. 2) The differentially expression fold change of 1.5 was filtered. and 3) The unique peptides of input gene should be >5. Volcano and heatmap were plotted using a free online platform for data analysis, the OmicShare tools (www.omicshare.com/tools). The mass spectrometry proteomics data have been deposited to the ProteomeXchange Consortium (http://proteomecentral.proteomexchange.org) via the PRIDE partner repository with the dataset identifier PXD028569 (8).

**RNA extraction and quantitative real-time PCR**

Total RNA of HRECs or lungs was isolated using Trizol (Transgen Biotech) and reverse transcribed with EasyScript One-Step RT-PCR SuperMix (TransGen Biotech). Quantitative real-time PCR was performed using ABI 7500 and TransStart Tip Green qPCR SuperMix (TransGen Biotech). The sets of primers were listed in supplementary table S5.

**HEK293 Cell culture and transfection**

HEK293T cells (ATCC, catalog # CRL-3216™) were maintained in DMEM (Gibco, catalog # 11995040, with 10% fetal bovine serum) and transfected with expression plasmid mixture (human wild-type or variant N-FLAG-*CTNND1* with or without VE-cadherin expression plasmid) by lipofectamine 3000 (Invitrogen, catalog # L3000015). Cells were collected 48 h later in gentle lysis buffer with protease and phosphatase inhibitor cocktails.

**Co-immunoprecipitation (co-IP) and Immunoblot analysis**

Co-IP and immunoblot analyses were conducted as previously described (2). Anti-FLAG Magnetic Beads (Med Chem Express, catalog # HY-K0207) was applied for co-immunoprecipitation with an incubation period for over-night at 4°C. The protein solutions were mixed with SDS-PAGE sample loading buffer and then loaded onto 12% Tris-glycine gels with following transfer onto nitrocellulose membranes. Cultured cells or tissues from mice were lysed in SDS lysis buffer (2% SDS and 62.5 mM Tris-HCl, pH 6.8, containing protease inhibitor cocktail tablets ordered from Roche, Inc.) and sonicated three times for 10s per time. Equal amounts of protein were loaded onto a 10% polyacrylamide gel and analyzed by immunoblotting. The primary antibodies used for immunoblotting were listed in supplementary Table S6.

**LiCl or CHIR-99021 treatment**

To rescue the down-regulated β-catenin signaling, *CTNND1-KD* HRECs were pretreated with 20 mM LiCl, 3 μM CHIR-99021 (Med Chem Express, catalog # HY-10182) or vehicle for 24 h. For LiCl treatment in mice, NaCl or LiCl (10 mg/kg, Sigma-Aldrich, catalog # L9650) dissolved in $ddH_2O$ were intraperitoneally injected daily in mice from P3 until the day of sacrifice.

**Supplemental Figures and Figure legends**

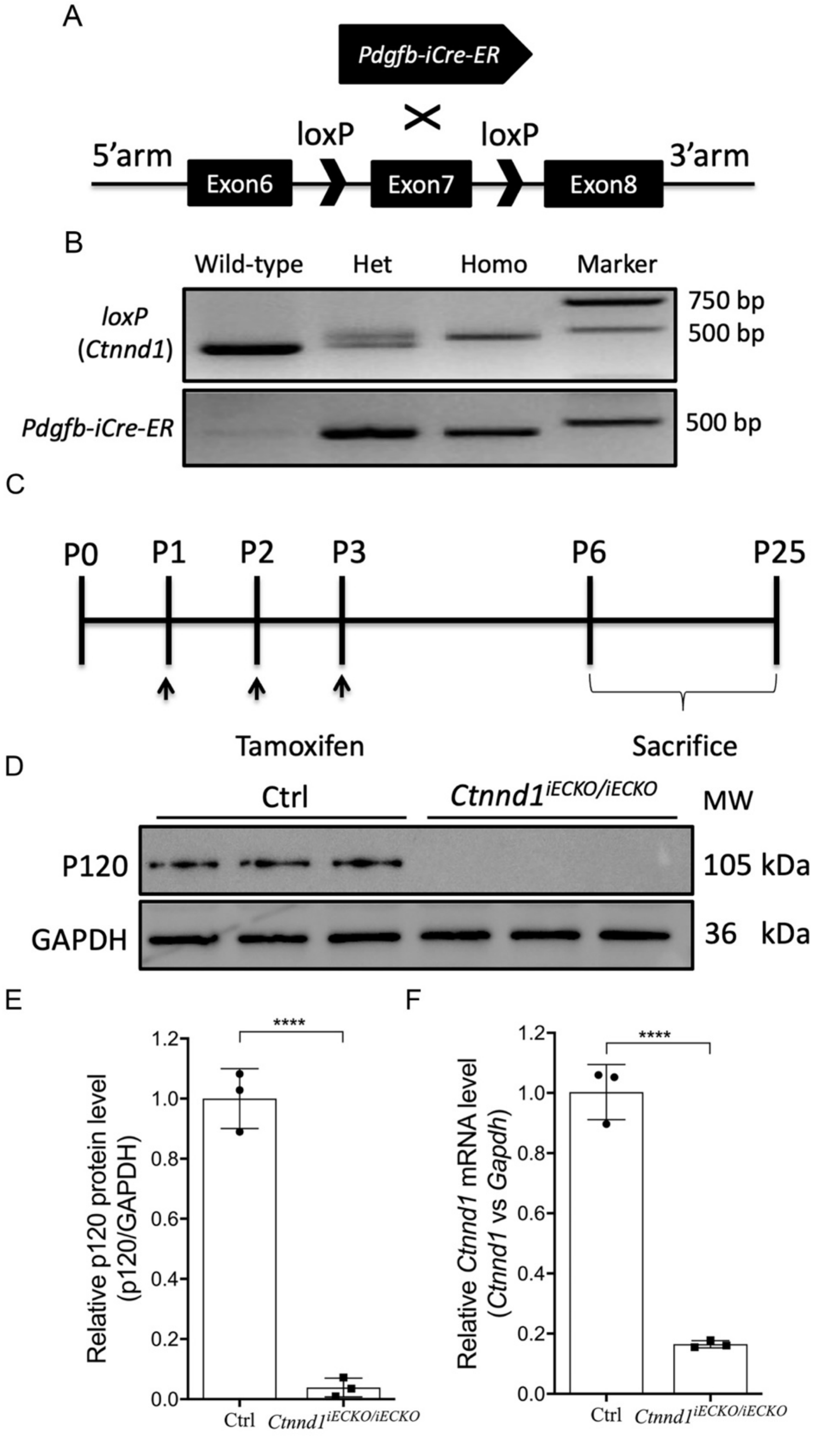


**Figure S1. Generation of EC-specific *Ctnnd1* knockout mice.**

(A) Schematic diagram for generation of *Ctnnd1*$^{iECKO/iECKO}$ mice. Exon 7 of *Ctnnd1* is flanked by loxP sites and was crossed to *pdgfb-iCre-ER*. (B) PCR amplification was carried out on genomic DNA to detect the presence of loxP sequences around *Ctnnd1* exon 7 and the *Cre* transgene. (C) Tamoxifen was intraperitoneally injected once

daily for consecutive three days at postnatal day 1 (P1), P2, P3 before sacrificing at P6~P25. (D) Western blot analysis of protein levels of p120 in P25 lung lysates of Ctrl and $Ctnnd1^{iECKO/iECKO}$ mice. (E) Quantification of relative p120 protein levels in P25 lung lysates of Ctrl and $Ctnnd1^{iECKO/iECKO}$ mice. Error bars, SDs. Student's t-test (n=3), **** p<0.0001. (F) Quantification of relative *Ctnnd1* mRNA levels detected by RT-qPCR in P25 Ctrl and $Ctnnd1^{iECKO/iECKO}$ mice lung. Error bars, SDs. Student's t-test (n=3), **** p<0.0001.

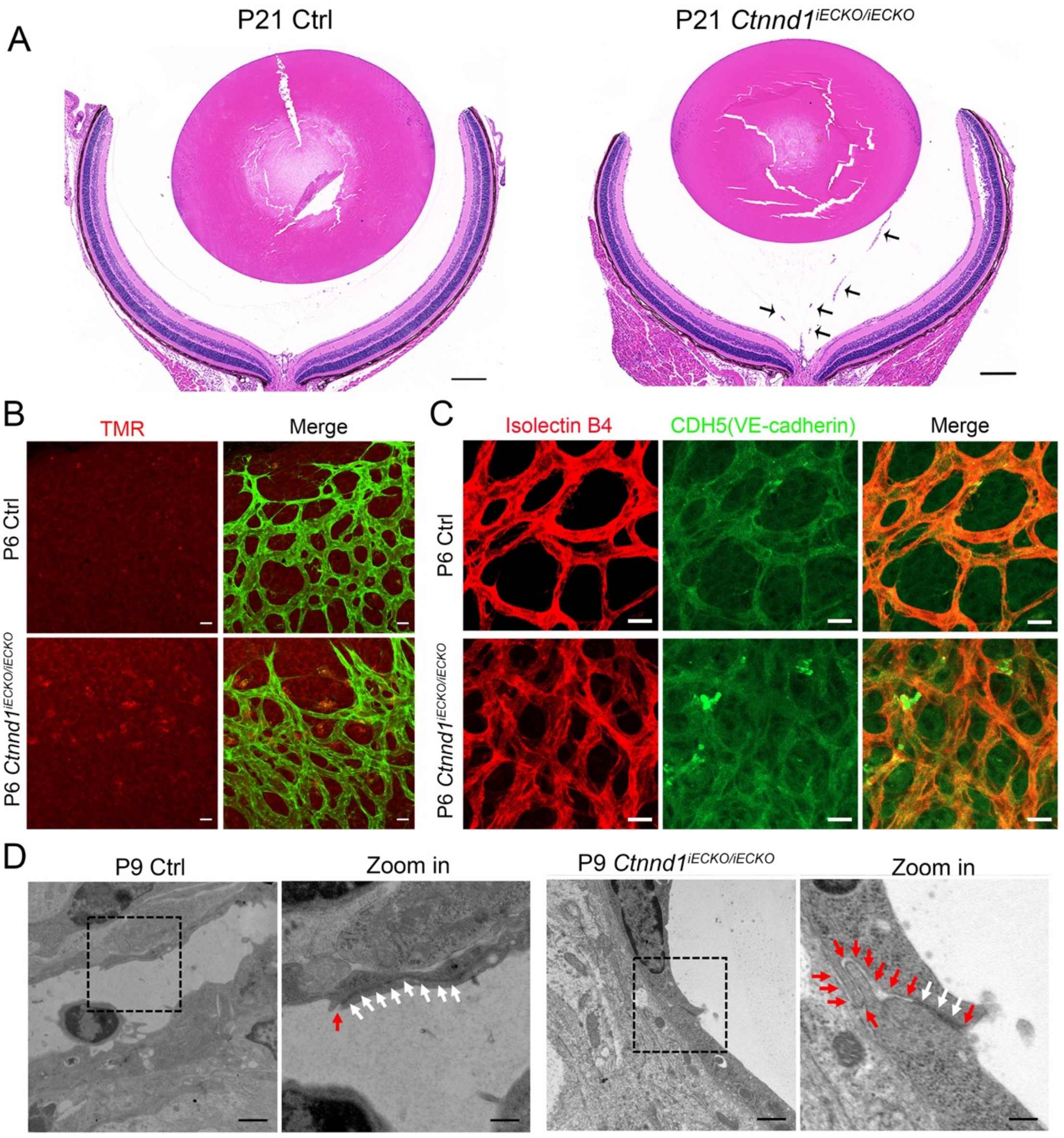


**Figure S2. Loss of *Ctnnd1* from mouse ECs inhibits hyaloid regression and disrupts junction integrity of retinal vessels.**

(A) Representative light micrographs of paraffin-embedded ocular sections from P21 Ctrl and *Ctnnd1$^{iECKO/iECKO}$* mice stained with hematoxylin and eosin. Scale bars, 200 µm. (B) Representative immunofluorescence images of biocytin-TMR (red) and IB4 (green) in the retinal flat mounts from P6 Ctrl and *Ctnnd1$^{iECKO/iECKO}$* mice. Scale bars, 20 µm. (C) Representative immunofluorescence images of P6 Ctrl and *Ctnnd1$^{iECKO/iECKO}$* mice retinas labeled with IB4 (red) and CDH5 (VE-cadherin, green). Scale bars, 20 µm. (D) Representative overview (left panels) and high-

magnification (right panels) electron microscope images of P9 Ctrl and *Ctnnd1*$^{iECKO/iECKO}$ mice retinas. Dotted boxes indicate magnified areas. Red and white arrows indicate discontinuous and continuous junctions between two adjacent ECs, respectively. Scale bars, 1μm (left panels) and 250 nm (right panels). Experiments were performed at least three times independently.

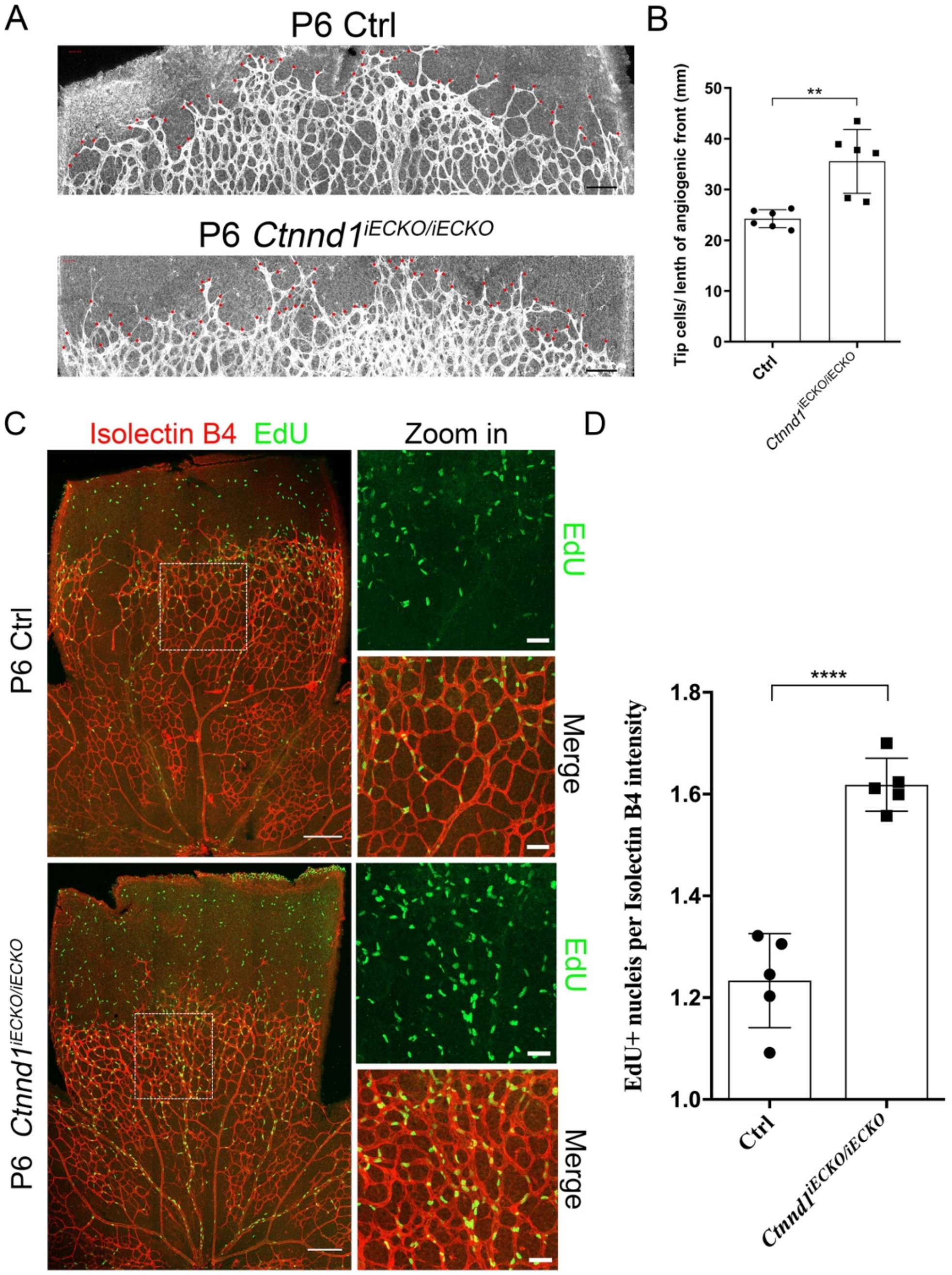


**Figure S3. Loss of *Ctnnd1* from mouse ECs promotes endothelial tip cell sprouting and peripheral endothelial proliferation.**

(A) Representative immunofluorescence images of P6 Ctrl and *Ctnnd1*$^{iECKO/iECKO}$ mouse retinas stained for IB4. The red "×" symbols indicate filopodia extending tip cells at the angiogenic front. Scale bars, 100 µm. (B) Quantification of the number of sprouts per unit of front length (mm) in P6 Ctrl and *Ctnnd1*$^{iECKO/iECKO}$ mice retinas. Error bars, SDs. Student's t-test (n=6), ** p<0.01. (C) Representative overview (left

panels) and high-magnification (right panels) immunofluorescence images of P6 Ctrl and *Ctnnd1*$^{iECKO/iECKO}$ mice retinas labeled with IB4 (red) and EdU (green). Scale bars, 250 µm (left panels) and 50 µm (right panels). (D) Quantification of EdU+ EC nucleus per IB4 intensity in the peripheral vasculature of P6 Ctrl and *Ctnnd1*$^{iECKO/iECKO}$ mice retinas. Error bars, SDs. Student's t-test (n=5), **** p<0.0001. Experiments were performed at least three times independently.

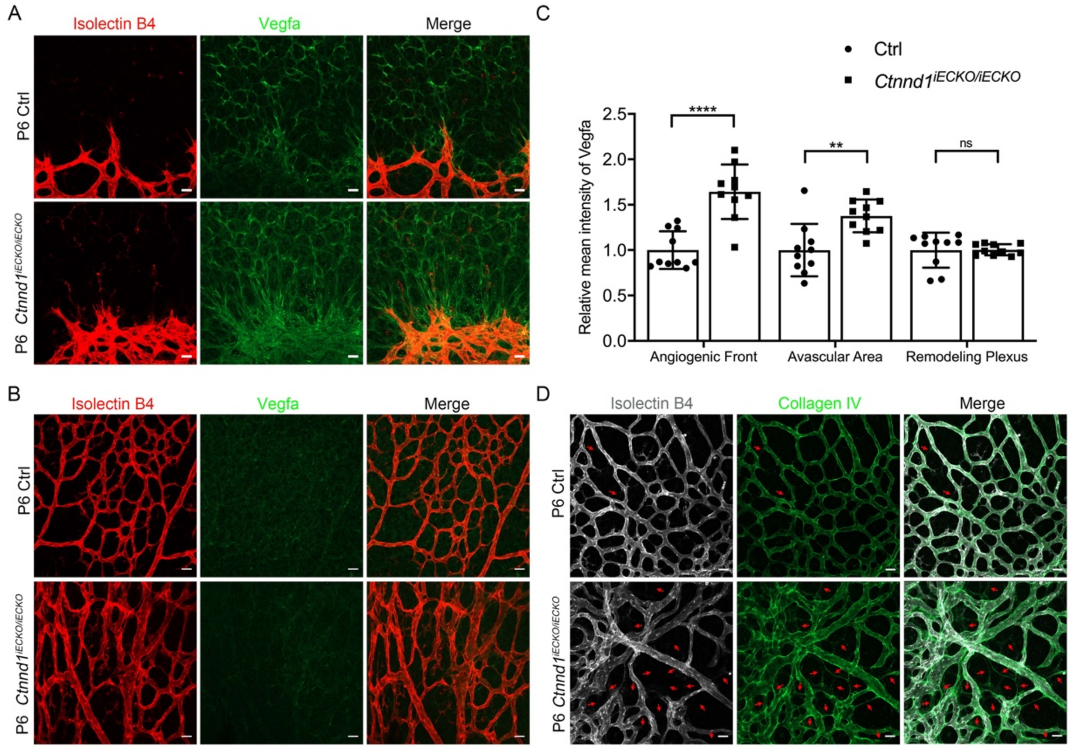


**Figure S4. Loss of *Ctnnd1* from mouse ECs promotes Vegfa expression and vessel pruning.**

(A) Representative immunofluorescence images of the peripheral retinal vasculature of P6 Ctrl and *Ctnnd1*$^{iECKO/iECKO}$ mice labeled with IB4 (red) and Vegfa (green). Scale bars, 20 µm. (B) Representative immunofluorescence images of the retinal remodeling plexus of P6 Ctrl and *Ctnnd1*$^{iECKO/iECKO}$ mice labeled with IB4 (red) and Vegfa (green). Scale bars, 20 µm. (C) Quantification of mean Vegfa intensity in the angiogenic front and avascular areas of P6 Ctrl and *Ctnnd1*$^{iECKO/iECKO}$ mice retinas. Error bars, SDs. Student's t-test (n=10), * p<0.05, **** p<0.0001. (D) Representative immunofluorescence images of P6 Ctrl and *Ctnnd1*$^{iECKO/iECKO}$ mouse retinas stained for IB4 (gray) and Collagen IV (green). Red arrows indicate Collagen IV$^{+}$, Isolectin B4$^{-}$ matrix sleeves. Scale bars, 20 µm. Experiments were performed at least three times independently.

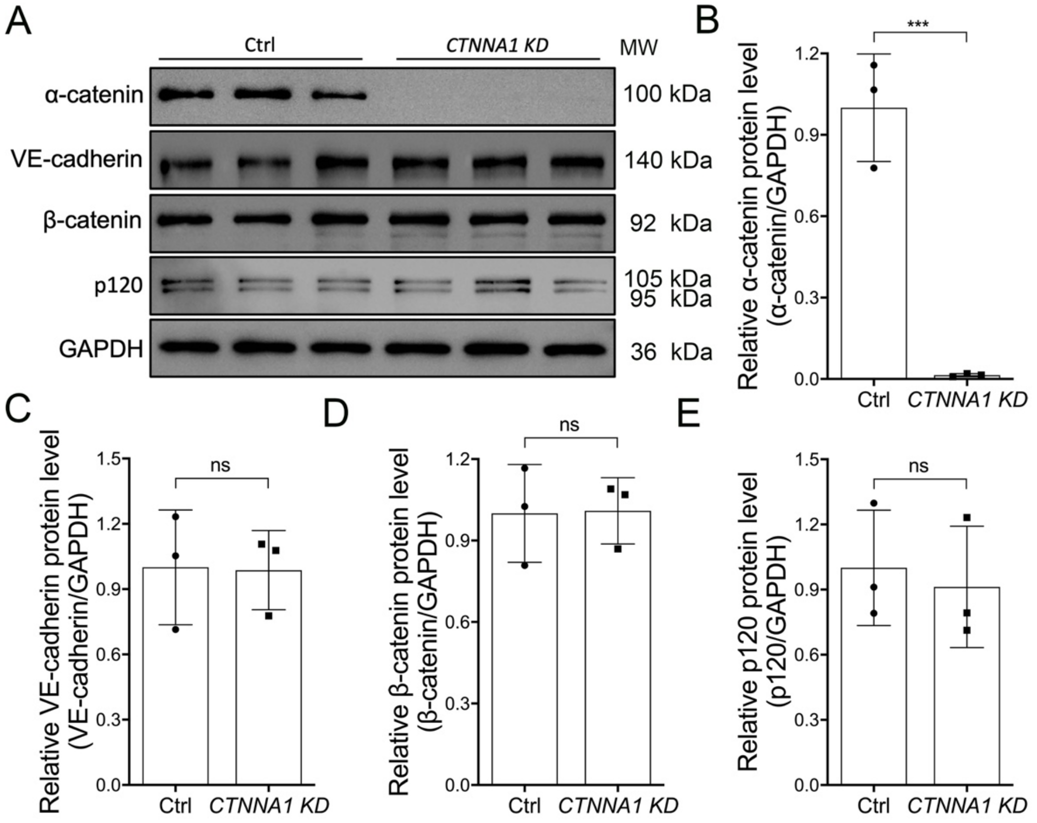


**Figure S5. The effect of *CTNNA1* knockdown on the protein levels of cadherin/catenin components.**

(A) Western blot analysis of protein levels of cadherin/catenin components (p120, α-catenin, β-catenin, and VE-cadherin) in Ctrl and *CTNNA1 KD* HRECs. (B) Quantification of relative p120, α-catenin, β-catenin, and VE-cadherin protein levels in Ctrl and *CTNNA1 KD* HRECs. Error bars, SDs. Student's t-test (n=3), ns, not significant, *** p<0.001. Experiments were performed at least three times independently.

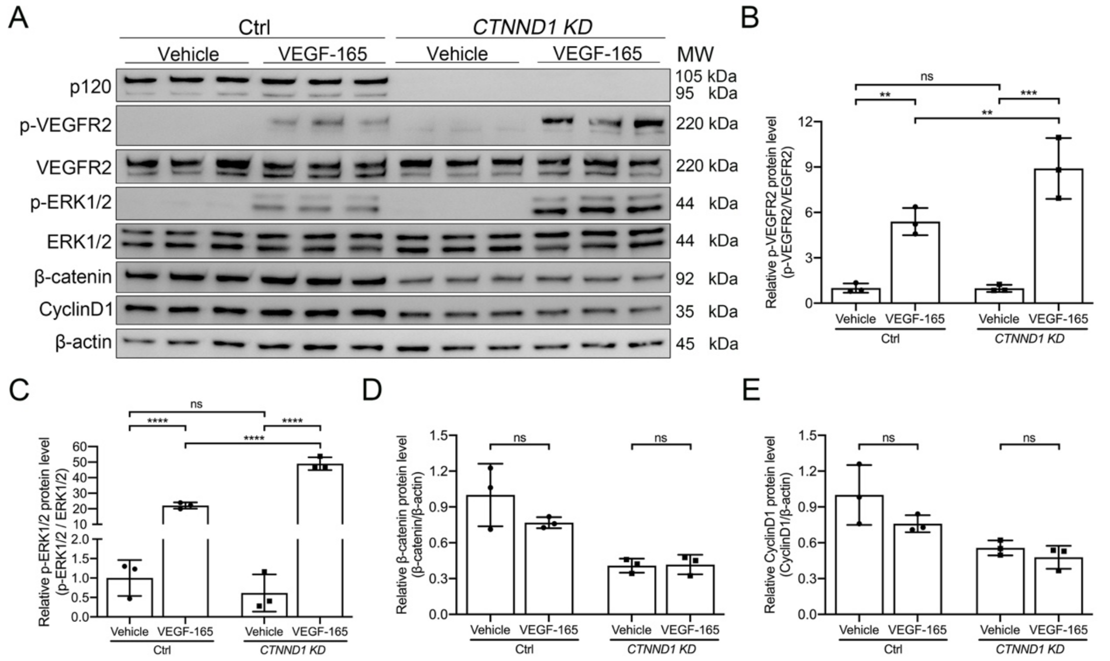


**Figure S6. VEGF responses in Ctrl and *CTNND1*-KD HRECs.**

(A) Western blot analysis of protein levels of p120, p-VEGFR2, p-ERK1/2, β-catenin, and CyclinD1 in Ctrl and *CTNND1 KD* HRECs treated with VEGF-165 or vehicle. (B-E) Quantification of relative protein levels of p-VEGFR2, p-ERK1/2, β-catenin, and CyclinD1 in Ctrl and *CTNND1 KD* HRECs treated with VEGF-165 or vehicle. Error bars, SDs. The p-values are from multiple comparisons in two-way ANOVA with Tukey's multiple comparisons test (n=3), ns, not significant, ** $p<0.01$, *** $p<0.001$, **** $p<0.0001$. Experiments were performed at least three times independently.

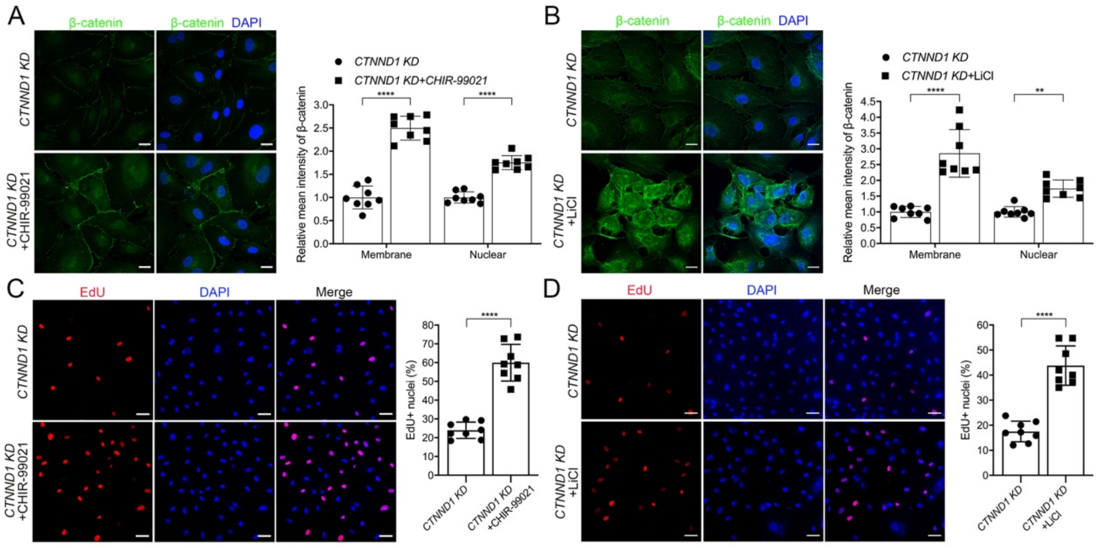


**Figure S7. LiCl or CHIR-99021 treatment restores β-catenin expression levels and enhances cell proliferation of *CTNND1 KD* HRECs.**

(A and B) Representative immunofluorescence images of β-catenin (green) and DAPI (blue) labeled *CTNND1 KD* HRECs, and quantification of relative mean intensity of membrane and nuclear-localized β-catenin in the *CTNND1 KD* HRECs treated with CHIR-99021 (A), LiCl (B) or vehicle. Scale bars, 20 µm. Error bars, SDs. Student's t-test (n=8), ** $p<0.01$, **** $p<0.0001$. (C and D) Representative immunofluorescence images of EdU (red) and DAPI (blue) labeled *CTNND1 KD* HRECs, and quantification of percentage of EdU+ nuclei in the *CTNND1 KD* HRECs treated with CHIR-99021 (C), LiCl (D) or vehicle. Scale bars, 50 µm. Error bars, SDs. Student's t-test (n=8), **** $p<0.0001$. Experiments were performed at least three times independently.

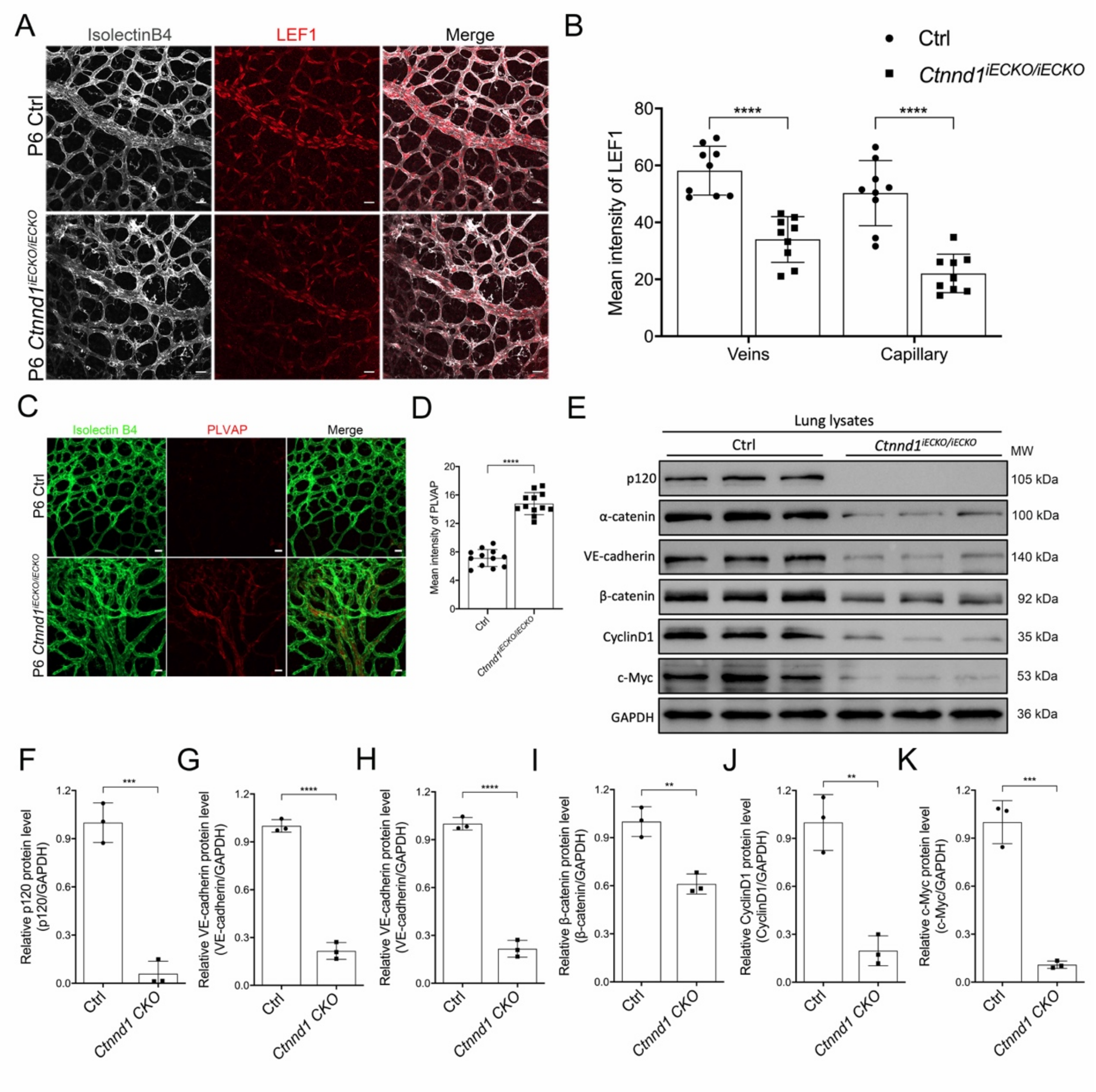

**Figure S8. Loss of *Ctnnd1* in mouse ECs inhibits Wnt signaling in the retinal ECs and lung lysates.**

(A) Representative immunofluorescence images of P6 Ctrl and *Ctnnd1*$^{iECKO/iECKO}$ mice retinas labeled with IB4 (gray) and LEF1 (red). Scale bars, 20 μm. (B) Quantification of mean LEF1 intensity in the veins and capillaries of P6 Ctrl and *Ctnnd1*$^{iECKO/iECKO}$ mice retinas. Error bars, SDs. Student's t-test (n=9), **** p<0.0001. (C) Representative immunofluorescence images of P6 Ctrl and *Ctnnd1*$^{iECKO/iECKO}$ mice retinas labeled with IB4 (green) and PLVAP (red). Scale bars, 20 μm. (D) Quantification of mean PLVAP intensity in the P6 Ctrl and *Ctnnd1*$^{iECKO/iECKO}$ mice retinas. Error bars, SDs. Student's t-test (n=12), **** p<0.0001. (E) Western blot analysis of p120, α-catenin, VE-cadherin, β-catenin, CyclinD1, and c-Myc protein levels in the total lung lysates of P25

Ctrl and *Ctnnd1*$^{iECKO/iECKO}$ mice. (F-K) Quantification of the relative protein levels of p120, α-catenin, VE-cadherin, β-catenin, CyclinD1, and c-Myc in the total lung lysates of P25 Ctrl and *Ctnnd1*$^{iECKO/iECKO}$ mice, Error bars, SDs. Student's t-test (n=3), ** p<0.01, *** p<0.001, **** p<0.0001. Experiments were performed at least three times independently.

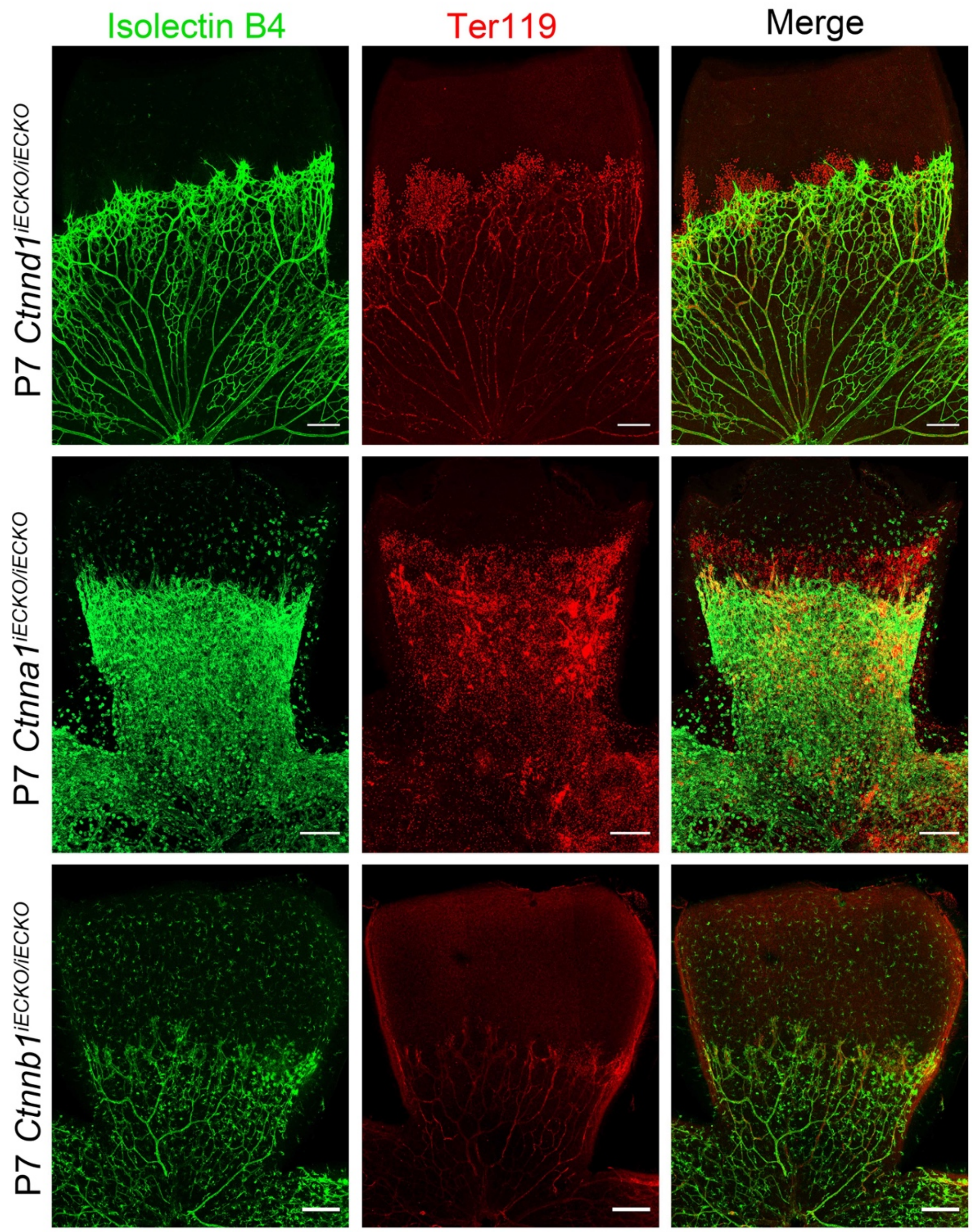


**Figure S9. Comparison of retina vasculature of EC-specific *Ctnnd1*, *Ctnna1* or *Ctnnb1* knockout mice.**

Representative immunofluorescence images of P7 *Ctnnd1*$^{iECKO/iECKO}$, *Ctnna1*$^{iECKO/iECKO}$, or *Ctnnb1*$^{iECKO/iECKO}$ mice retinas labeled with IB4 (green) and Ter119 (red). Scale bars, 200 μm. Experiments were performed at least three times independently.

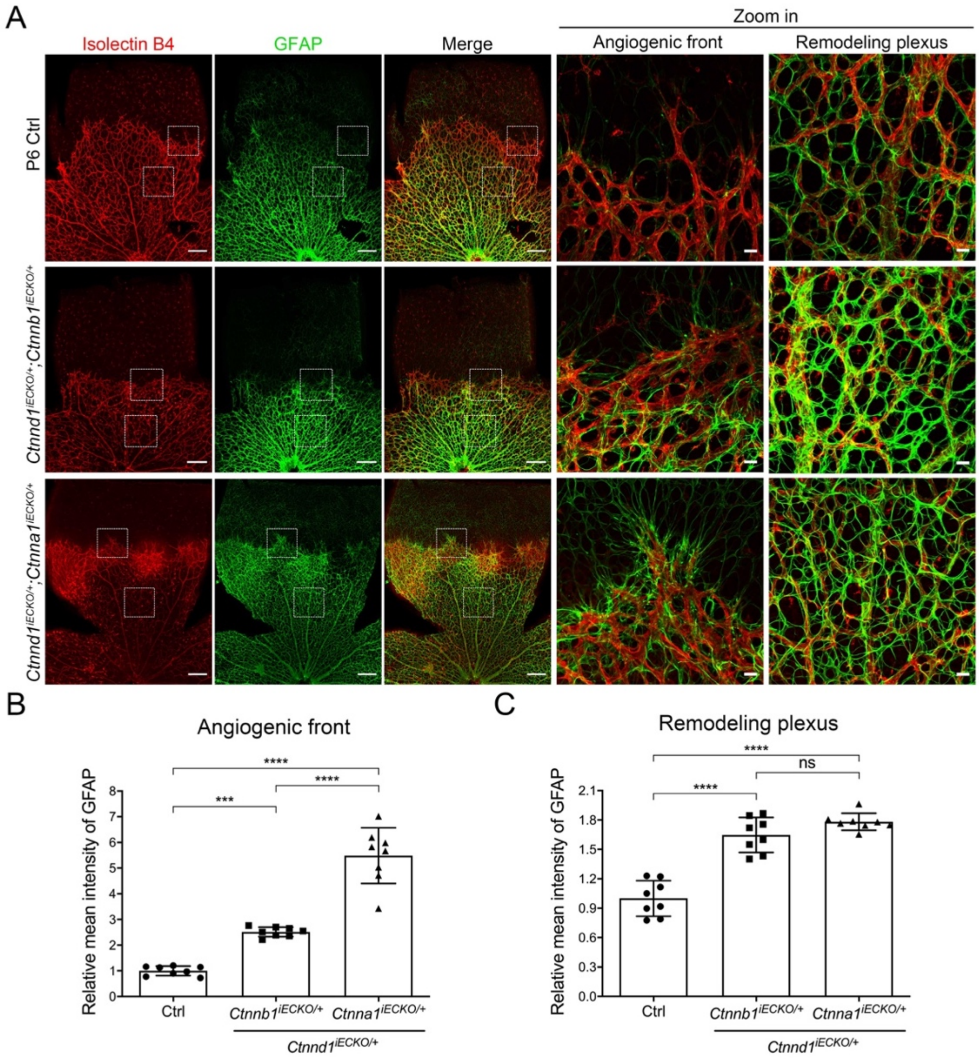


**Figure S10. Double heterozygous deletion of *Ctnnd1* and *Ctnna1* or *Ctnnb1* in mouse ECs lead to astrogliosis.**

(A) Representative overview and high-magnification immunofluorescence images of P6 Ctrl, *Ctnnd1*$^{iECKO/+}$; *Ctnnb1*$^{iECKO/+}$, and *Ctnnd1*$^{iECKO/+}$; *Ctnna1*$^{iECKO/+}$ mouse retinas labeled with IB4 (red) and GFAP (green). Dotted boxes indicate magnified areas in the angiogenic front and remodeling plexus. Scale bars, 200 μm and 20 μm. (B and C) Quantification of relative GFAP mean intensity in the angiogenic front (B) and remodeling plexus (C) of P6 Ctrl, *Ctnnd1*$^{iECKO/+}$; *Ctnnb1*$^{iECKO/+}$, and *Ctnnd1*$^{iECKO/+}$; *Ctnna1*$^{iECKO/+}$. Error bars, SDs. The p-values are from multiple comparisons in one-way ANOVA with Tukey's multiple comparisons test (n=8), ns, not significant, *** p<0.001, **** p<0.0001. Experiments were performed at least three times independently.

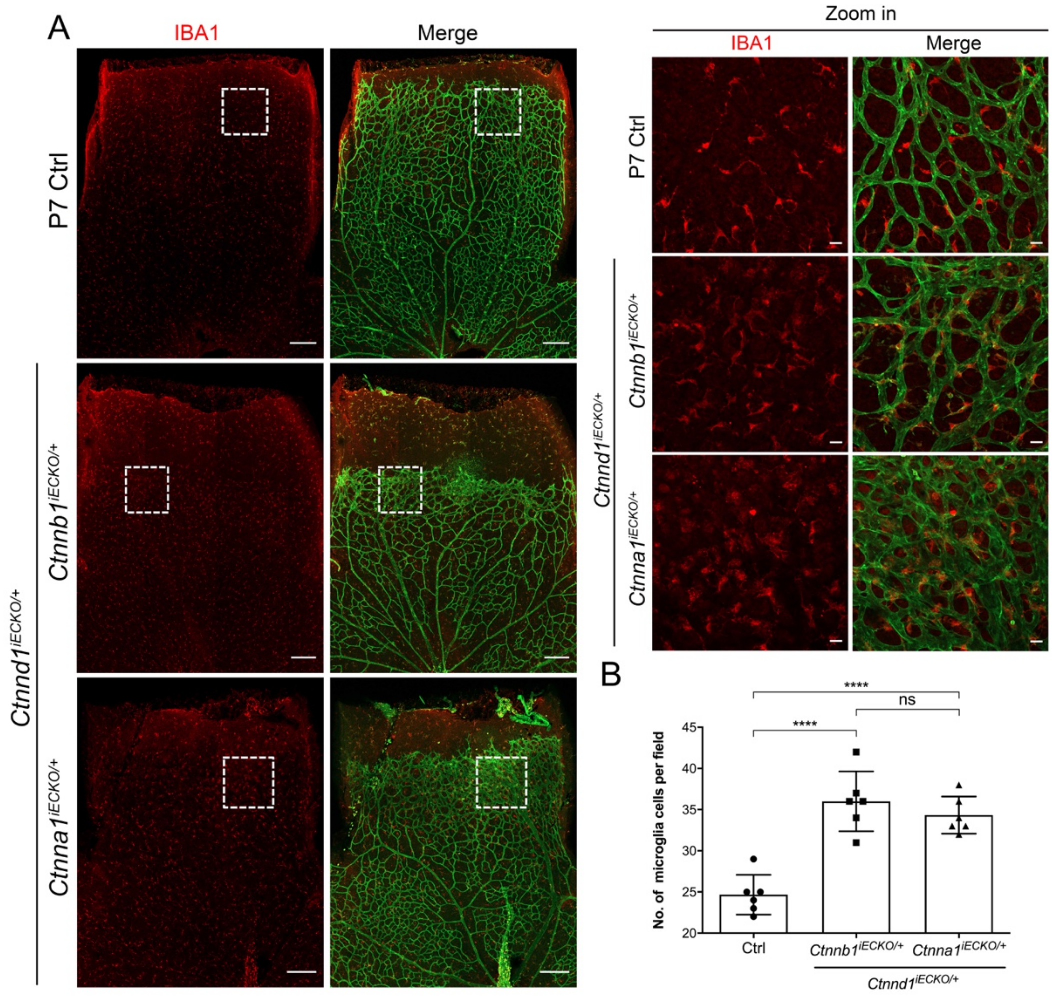


**Figure S11. Double heterozygous deletion of *Ctnnd1* and *Ctnna1* or *Ctnnb1* in mouse ECs lead to microgliosis.**

(A) Representative overview and high-magnification immunofluorescence images of P7 Ctrl, *Ctnnd1*$^{iECKO/+}$; *Ctnnb1*$^{iECKO/+}$, and *Ctnnd1*$^{iECKO/+}$; *Ctnna1*$^{iECKO/+}$ mouse retinas labeled with IB4 (green) and IBA1 (red). Dotted boxes indicate magnified areas. Scale bars, 200 µm and 20 µm. (B) Quantification of number of IBA1-expressing microglia in P7 Ctrl, *Ctnnd1*$^{iECKO/+}$; *Ctnnb1*$^{iECKO/+}$, and *Ctnnd1*$^{iECKO/+}$; *Ctnna1*$^{iECKO/+}$ mouse retinas. Error bars, SDs. The p-values are from multiple comparisons in one-way ANOVA with Tukey's multiple comparisons test (n=6), ns, not significant, **** p<0.0001. Experiments were performed at least three times independently.

**Table S2. Primers for Sanger sequencing**

| Primer name | Primers (5'-3') |
|---|---|
| *CTNND1* c.949C>T (p.R317C)-F | GTATAGGCCCAGCATGGAAG |
| *CTNND1* c.949C>T (p.R317C)-R | GCCAAGTCAGAAAAGGGAAA |
| *CTNND1* c.1867A>T (p.K623*)-F | ATTTCAAGAAGCCTGGAGCA |
| *CTNND1* c.1867A>T (p.K623*)-R | CAAAGGACGTATGGGGAAGA |
| *CTNND1* c.2099G>A (p.R700Q)-F | GCCAGCTAGAGCCTGGTTTA |
| *CTNND1* c.2099G>A (p.R700Q)-R | GAGGGTCCTTCCCTTCAGTC |

**Table S3. Primers for mouse genotyping**

| Primer name | Primers (5’-3’) |
|---|---|
| *Ctnnd1*$^{flox}$-F | AGGGAGAGAGTTCAGTTGGTGAAATG |
| *Ctnnd1*$^{flox}$-R | CCTCTTCACCAATCATGTCTTCATAGCT |
| *Ctnna1*$^{flox}$-F | CATTTCTGTCACCCCCAAAGACAC |
| *Ctnna1*$^{flox}$-R | GCAAAATGATCCAGCGTCCTGGG |
| *Ctnnb1*$^{flox}$-F | AAGGTAGAGTGATGAAAGTTGTT |
| *Ctnnb1*$^{flox}$-R | CACCATGTCCTCTGTCTATTC |
| *Pdgfb-iCre-ER*-F | GCCGCCGGGATCACTCTCG |
| *Pdgfb-iCre-ER*-R | CCAGCCGCCGTCGCAACTC |

**Table S4. Antibodies for Immunohistochemistry**

| Antibodies | Dilution ratio; Catalog #; Brand |
|---|---|
| Isolectin GS-IB4 Alexa Fluor™ 594 Conjugate | 1:100 dilution; I21413; Invitrogen |
| Isolectin GS-IB4 Alexa Fluor™ 488 Conjugate | 1:100 dilution; I21411; Invitrogen |
| Monoclonal Anti-FLAG® M2 antibody produced in mouse | 1:100 dilution; F3165; Sigma-Aldrich |
| rat anti-mouse Ter-119 | 1:50 dilution; 553670; BD Bioscience |
| rabbit anti-VE-Cadherin | 1:100 dilution; 2500; Cell Signaling Technology |
| rat anti-mouse VE-Cadherin | 1:100 dilution; 555289; BD Bioscience |
| goat anti-mouse ACTA2 | 1:100 dilution; AF1999; R&D Systems |
| rabbit anti-GFAP | 1:100 dilution; 12389; Cell Signaling Technology |
| goat anti-mouse Vegf164 | 1:100 dilution; AF-493-NA; R&D Systems |
| mouse anti-alpha catenin | 1:100 dilution; 13-9700; Thermo Fisher Scientific |
| rabbit anti-CTNNB1 | 1:100 dilution; 9582; Cell Signaling Technology |
| rabbit anti-CTNND1 | 1:100 dilution; 59854; Cell Signaling Technology |
| rabbit anti-LEF1 | 1:100 dilution; 76010; Cell Signaling Technology |
| rat anti-mouse PLVAP | 1:50 dilution; 553849; BD Biosciences |
| Rabbit anti-IBA1 | 1:100 dilution; 019-19741; Wako |
| DAPI | 1:2000 dilution; 4083; Cell Signaling Technology |
| goat anti-mouse IgG (H+L), Alexa Fluor Plus 488 | 1:500 dilution; A32723; Invitrogen |

| | |
|---|---|
| goat anti-rat IgG (H+L), Alexa Fluor™-488 | 1:500 dilution; A-11006; Invitrogen |
| donkey anti-goat IgG (H+L), Alexa Fluor™-488 | 1:500 dilution; A32814; Invitrogen |
| goat anti-rabbit IgG (H+L), Alexa Fluor™-488 | 1:500 dilution; A32721; Invitrogen |
| goat anti-rabbit IgG (H+L), Alexa Fluor™-594 | 1:500 dilution; A32740; Invitrogen |

**Table S5. Primers for QPCR**

| Primer name | Primers (5'-3') |
|---|---|
| Human-GAPDH-F | CTCTGCTCCTCCTGTTCGAC |
| Human-GAPDH-R | TTAAAAGCAGCCCTGGTGAC |
| Human-CTNNA1- F | GCGAATTGTGGCAGAGTGTA |
| Human-CTNNA1- R | GCAAGTCCCTGGTCTTCTTG |
| Human-CTNND1- F | ATGGGCTATGATGACCTGGA |
| Human-CTNND1- R | CAGCTCTGGCTGTCTCCAAT |
| Human-CDH5- F | GCTGGTCACTCTGCAAGACA |
| Human-CDH5- R | TCATCTGGGTCCTCAACAAA |
| Human-CLDN5- F | AAAGAGATCCCCCTGCATTT |
| Human-CLDN5- R | GTGAGCATCTCCTCCGAGAC |
| Human-CCND1- F | TGAGGCGGTAGTAGGACAGG |
| Human-CCND1- R | GACCTTCGTTGCCCTCTGT |
| Human-MYC- F | CACCGAGTCGTAGTCGAGGT |
| Human-MYC- R | TTTCGGGTAGTGGAAAACCA |
| Human-CTNNB1- F | GTGGACCACAAGCAGAGTGC |
| Human-CTNNB1- R | TAGTTGCAGCATCTGAAAGATTCC |
| Human-DKK1-F | ATGCGTCACGCTATGTGCT |
| Human-DKK1-R | TCTGGAATACCCATCCAAGG |
| Human-JUN-F | AGGAGGAGCCTCAGACAGTG |
| Human-JUN-R | AGCTTCCTTTTTCGGCACTT |

| | |
|---|---|
| Human-CD44-F | CACGTGGAATACACCTGCAA |
| Human-CD44-R | GACAAGTTTTGGTGGCACG |
| Mouse-Ctnnd1-F | GACCTCGATTACGGCATGAT |
| Mouse-Ctnnd1-R | CCGCTCATGCTGAGCTAAAG |
| Mouse-Gapdh-F | TGTGTCCGTCGTGGATCTGA |
| Mouse-Gapdh-R | TTGCTGTTGAAGTCGCAGGAG |

**Table S6. Antibodies for Western blots**

| Antibodies | Dilution ratio; Catalog #; Brand |
|---|---|
| Monoclonal Anti-FLAG® M2 antibody produced in mouse | 1:100 dilution; F3165; Sigma-Aldrich |
| rabbit anti-VE-Cadherin | 1:1000 dilution; 2500; Cell Signaling Technology |
| rabbit anti-CTNNB1 | 1:1000 dilution; 9582; Cell Signaling Technology |
| rabbit anti-CTNND1 | 1:1000 dilution; 59854; Cell Signaling Technology |
| rabbit anti-GLUT1 | 1:1000 dilution; A11727; Abclonal |
| rabbit anti-Cyclind1 | 1:1000 dilution; MA5-14512; Thermo Fisher |
| rabbit anti-c-Myc | 1:1000 dilution; 10828-1-AP; Proteintech |
| rabbit anti-β-actin | 1:1000 dilution; 20536-1-AP; Proteintech |
| rabbit anti-GAPDH | 1:1000 dilution; 60004-1-Ig; Proteintech |
| anti-mouse IgG, HRP-linked Antibody | 1:10000 dilution; 7076; Cell Signaling Technology |
| anti-rabbit IgG, HRP-linked Antibody | 1:10000 dilution; 7074; Cell Signaling Technology |

## References

1. National Genomics Data Center M, and Partners. Database Resources of the National Genomics Data Center in 2020. *Nucleic Acids Res.* 2020;48(D1):D24-D33.
2. Zhu X, Yang M, Zhao P, Li S, Zhang L, Huang L, et al. Catenin alpha 1 mutations cause familial exudative vitreoretinopathy by overactivating Norrin/beta-catenin signaling. *The Journal of clinical investigation.* 2021;131(6).
3. Pitulescu ME, Schmidt I, Benedito R, and Adams RH. Inducible gene targeting in the neonatal vasculature and analysis of retinal angiogenesis in mice. *Nature protocols.* 2010;5(9):1518-34.
4. He Y, Yang M, Zhao R, Peng L, Dai E, Huang L, et al. Novel truncating variants in CTNNB1 cause familial exudative vitreoretinopathy. *J Med Genet.* 2022.
5. Meier F, Brunner AD, Frank M, Ha A, Bludau I, Voytik E, et al. diaPASEF: parallel accumulation-serial fragmentation combined with data-independent acquisition. *Nature methods.* 2020;17(12):1229-36.
6. Cui Y, Liu P, Mooney BP, and Franz AWE. Quantitative Proteomic Analysis of Chikungunya Virus-Infected Aedes aegypti Reveals Proteome Modulations Indicative of Persistent Infection. *Journal of proteome research.* 2020;19(6):2443-56.
7. Mi H, Muruganujan A, Ebert D, Huang X, and Thomas PD. PANTHER version 14: more genomes, a new PANTHER GO-slim and improvements in enrichment analysis tools. *Nucleic Acids Res.* 2019;47(D1):D419-D26.
8. Perez-Riverol Y, Csordas A, Bai J, Bernal-Llinares M, Hewapathirana S, Kundu DJ, et al. The PRIDE database and related tools and resources in 2019: improving support for quantification data. *Nucleic Acids Res.* 2019;47(D1):D442-D50.
